# Division of Labor Between Two Actin Nucleators—the Formin FH1 and the ARP2/3 Complex—in *Arabidopsis* Epidermal Cell Morphogenesis

**DOI:** 10.3389/fpls.2020.00148

**Published:** 2020-03-02

**Authors:** Petra Cifrová, Denisa Oulehlová, Eva Kollárová, Jan Martinek, Amparo Rosero, Viktor Žárský, Kateřina Schwarzerová, Fatima Cvrčková

**Affiliations:** ^1^ Department of Experimental Plant Biology, Faculty of Science, Charles University, Prague, Czechia; ^2^ Institute of Experimental Botany, Academy of Sciences of the Czech Republic, Prague, Czechia

**Keywords:** actin nucleation, ARP2/3, At3g25500, At4g01710, cytoskeleton, formin, pavement cell, trichome

## Abstract

The ARP2/3 complex and formins are the only known plant actin nucleators. Besides their actin-related functions, both systems also modulate microtubule organization and dynamics. Loss of the main housekeeping *Arabidopsis thaliana* Class I membrane-targeted formin FH1 (At3g25500) is known to increase cotyledon pavement cell lobing, while mutations affecting ARP2/3 subunits exhibit an opposite effect. Here we examine the role of FH1 and the ARP2/3 complex subunit ARPC5 (At4g01710) in epidermal cell morphogenesis with focus on pavement cells and trichomes using a model system of single *fh1* and *arpc5*, as well as double *fh1 arpc5* mutants. While cotyledon pavement cell shape in double mutants mostly resembled single *arpc5* mutants, analysis of true leaf epidermal morphology, as well as actin and microtubule organization and dynamics, revealed a more complex relationship between the two systems and similar, rather than antagonistic, effects on some parameters. Both *fh1* and *arpc5* mutations increased actin network density and increased cell shape complexity in pavement cells and trichomes of first true leaves, in contrast to cotyledons. Thus, while the two actin nucleation systems have complementary roles in some aspects of cell morphogenesis in cotyledon pavement cells, they may act in parallel in other cell types and developmental stages.

## Introduction

The shape of plant cells (and subsequently also organs and tissues) is mainly controlled by orchestrated action of microfilaments, microtubules, and membrane trafficking in the cortical cytoplasm, resulting in spatially and temporally controlled cell growth and cell wall synthesis. Cortical microtubules direct cellulose deposition (see Paredez et al., 2006; Bashline et al., 2014), microfilaments participate in exocytosis (e.g. Sampathkumar et al., 2013), and actomyosin-driven cytoplasmic streaming also contributes to cell expansion (Peremyslov et al., 2015). The cortical cytoplasm also controls localization of membrane proteins including auxin transporters, contributing thus to cell differentiation and affecting tissue- and organ-scale developmental processes (e.g., Žárský et al., 2009; Qi and Greb, 2017). Microfilament organization and dynamics are regulated by numerous proteins, including *de novo* actin nucleators. Formins and the ARP2/3 complex are the only two actin-nucleating systems found so far both in plants and opisthokonts, representing thus conserved molecular mechanisms inherited from the common eukaryotic ancestor (e.g. Vaškovičová et al., 2013).

Formins share the conserved FH2 domain whose dimer can nucleate and cap actin filaments, usually accompanied by a profilin-G-actin-binding FH1 domain and by additional domains mediating regulatory or structural interactions that vary both within and between lineages. Angiosperms have two clades of formins consisting of multiple paralogs, with over 20 genes in *Arabidopsis* (Grunt et al., 2008). Besides their actin-related roles, formins contribute to the coordination between microfilaments and microtubules (Bartolini and Gundersen, 2010; Wang et al., 2012; Henty-Ridilla et al., 2016). Binding of formins to microtubules has been documented also in plants (Deeks et al., 2010; Li et al., 2010; Yang et al., 2011; Wang et al., 2013). Some formins are associated with membranes and modulate endomembrane dynamics (see Gurel et al., 2014; Cvrčková et al., 2014). Typical plant Class I formins are transmembrane proteins that can anchor cytoskeletal structures to the plasmalemma, its distinct domains, and/or endomembranes (e.g., Deeks et al., 2010; Martinière et al., 2011; Diao et al., 2018; Oulehlová et al., 2019). Plant Class II formins typically harbor a Phosphatase and Tensin (PTEN)-like domain implicated in phospholipid binding and membrane localization (van Gisbergen et al., 2012). Direct or interactor-mediated membrane association, or role in endomembrane organization, is documented also for some opisthokont formins lacking membrane insertion motifs (reviewed in Cvrčková, 2013; see, e.g., Copeland et al., 2016).

Mutations affecting the main *Arabidopsis* housekeeping Class I formin, FH1, or pharmacological inhibition of formin function by the SMIFH2 compound, have only minor phenotypic consequences that include increased pavement cell and trichome shape complexity, but a profound impact on both actin and microtubule organization and dynamics (Rosero et al., 2013; Rosero et al., 2016; Cvrčková and Oulehlová, 2017; Oulehlová et al., 2019). Changes in microtubule organization were also reported for mutants of the rice microtubule-binding Class II formin FH5 (Yang et al., 2011; Zhang Z. et al., 2011).

The other evolutionarily conserved actin nucleation system found in plants, the ARP2/3 complex, comprises two actin-related proteins (ARP2 and ARP3) and five additional conserved subunits termed ARPC1-5. Some subunits might be dispensable in specific cellular contexts (see Pizarro-Cerdá et al., 2017). Upon activation by regulatory complexes termed the NPFs (nucleation promoting factors), which exhibit considerable diversity across eukaryotes (Dominguez, 2016), the ARP2/3 complex mediates nucleation of new actin filaments (see e.g. Rotty et al., 2013; Yanagisawa et al., 2013). Characteristic for ARP2/3-initiated filament arrays is their branching angle of about 70°, also documented in plants (Fišerová et al., 2006). Like formins, the ARP2/3 complex also has roles outside controlling actin dynamics. In metazoans, it can associate with microtubule-nucleating gamma tubulin complexes (Hubert et al., 2011) and some NPFs bind to microtubules and endomembranes (Campellone et al., 2008). Plant ARPC4 and ARPC2 localize to microtubules, with the later binding them also *in vitro* (Zhang et al., 2013b; Havelková et al., 2015). ARPC4 is associated with endomembrane compartments and the NPF complex subunit NAP1 localizes to the endoplasmic reticulum (Yanagisawa et al., 2013; Zhang et al., 2013a), as well as autophagosomes (Wang et al., 2016).

*Arabidopsis* mutations affecting the ARP2/3 complex function and regulation result in typical “distorted” trichome phenotype and reduced pavement cell lobing (Mathur et al., 2003a, see Ivakov and Persson, 2013; Sahi et al., 2018), as well as altered microtubule organization (Saedler et al., 2004; Zhang et al., 2005). Mutants also exhibit changes in cell wall composition, although the responsible mechanism remains to be characterized (Sahi et al., 2018).

Relations between the formins and the ARP2/3 complex are so far poorly understood. Formins appear to generate actin bundles, while the ARP2/3 complex produces fine, branched microfilament arrays (see Carlier and Shekhar, 2017). Coordination of the two actin nucleation systems may be ensured by several possible mechanisms. The balance between ARP2/3 and formin-driven actin assembly in some metazoan cell types and fission yeast may involve the profilin/G-actin ratio or competition for G-actin monomers bound to profilin (Rotty et al., 2015; Suarez et al., 2015). While profilin-dependent, formin-mediated actin nucleation is limiting for the rate of *Arabidopsis* epidermal cells elongation (Cao et al., 2016), the relevance of competition between the two nucleation mechanisms for free or profilin-bound G-actin is questionable in plants, which generally have a higher ratio of free to polymerized actin than opisthokonts (Blanchoin et al., 2010). Another possible shared regulatory mechanism involves the heterodimeric capping protein, which controls the availability of barbed ends and participates in the control of actin dynamics in both opisthokonts (Edwards et al., 2014) and plants (Pleskot et al., 2012; Jimenez-Lopez et al., 2014; Li et al., 2014). In opisthokonts, the two actin nucleation systems also share regulatory inputs from RHO clade small GTPases (Liu et al., 2009). Plant-specific RHO GTPases, ROPs, control cell morphogenesis including pavement cell lobing (Fu et al., 2002). However, since plant formins lack the GBD/FH3 small GTPase-interacting domain, common in the opisthokonts (Grunt et al., 2008), the actual regulatory mechanism must differ between opisthokonts and plants. The two actin nucleating systems may also share interactors, including regulatory ones. Common interactors, or at least genetic interaction, of formins and ARP2/3 or the NPF complexes, were also found in fission yeast (Carnahan and Gould, 2003) and Drosophila (Liu et al., 2009), and mammalian formins and ARP2/3 complex subunits co-localize in tight junctions (Grikscheit and Grosse, 2016). Thus, while the existence of numerous interactions and/or co-ordination between ARP2/3 and formins is likely, a consistent model of the “division of labor” between these two ancient actin nucleation systems is still lacking even in opisthokonts.

In this study, we characterize the effects of mutational inactivation of genes encoding the main housekeeping Class I formin of *Arabidopsis* vegetative tissues, FH1 (At3g25500), one of the ARP2/3 complex subunits, ARPC5 (At4g01710), or both genes simultaneously, on epidermal cell morphogenesis and cytoskeletal organization and dynamics. Both *fh1* and *arpc5* mutations were previously shown to cause opposite alterations in epidermal pavement cell shape, i.e. the *arpc5* mutation, as well as FH1 overexpression, led to a decrease and *fh1* mutation to an increase in pavement cell lobing (Mathur et al., 2003b; Li et al., 2003; Rosero et al., 2016; Sahi et al., 2018; Oulehlová et al., 2019). The layout of pavement cell lobes is determined by reorganization of cortical microtubules prior to lobe emergence, while the role of microfilaments in this process remains controversial (Panteris and Galatis, 2005; Armour et al., 2015). In addition, *ARPC5* is also identical to *CROOKED* (*CRK*), a gene whose mutation resulted in trichome deformation characteristic for the distorted class mutants (Mathur et al., 2003b). Remarkably, overexpression of GFP-tagged FH1 results in a distorted trichome phenotype, while loss-of-function *fh1* mutants exhibit an increase in trichome branch number (Oulehlová et al., 2019), consistent with possible antagonistic function of FH1 and the Arp2/3 complex in trichome development.

Based on these known phenotypes of *fh1* and *arpc5* mutants, we hypothesized that the two actin nucleation systems might play opposite roles in pavement cell, and possibly trichome, shaping. This would lead to the prediction that double *fh1 arpc5* mutants might, in part or completely, revert to a phenotype closer to the wild type (wt) than either mutation alone. Though our observations do not support this hypothesis, they indicate a more complex relationship between the two systems and reveal similar, rather than antagonistic, effects on some parameters. We also documented unexpected differences between the mutant phenotypes in cotyledons and true leaves, suggesting a developmental stage-dependent relationship between the two actin nucleation systems.

## Materials and Methods

### Plants

The following *Arabidopsis thaliana* T-DNA insertional mutants obtained through The Nottingham Arabidopsis Stock Centre (NASC) have been used in this study. For the *FH1* (At3g25500) gene, we employed *fh1-1* (SALK-032981), one of the two alleles used to establish the effects of *fh1* mutation on cytoskeletal organization and dynamics in our previous work (Rosero et al., 2013; Rosero et al., 2016) and *fh1-4* (SALK-N551065), shown to affect epidermal cell shape in the same manner as *fh1-1* (Oulehlová et al., 2019). Both alleles were successfully complemented by expression of GFP-tagged wt FH1 protein at near-native levels (Oulehlová et al., 2019). For the *ARPC5* (At4g01710) gene, we used the *arpc5-1* (SALK-123936) allele previously documented to produce epidermal phenotypic deviations indistinguishable from those caused by a recessive EMS-generated mutation in the same gene, *crk-1* (Li et al., 2003; Mathur et al., 2003b; see also Mathur, 2005). In some experiments, previously characterized T-DNA insertion mutants *arp2* (SALK-077920) and *arpc4* (SALK-013909) have been employed (Sahi et al., 2018). The trichome morphogenesis defect of *arpc5-1* mutants was fully complemented by expression of GFP-tagged wt ARPC5 (J. Martinek and K. Schwarzerová, unpublished). For the purpose of line verification and in subsequent crosses, the allelic status of T-DNA insertions was determined by PCR as reported in our previous studies (Rosero et al., 2013; Sahi et al., 2018; Oulehlová et al., 2019).

The *fh1*:CRISPR mutant was generated as follows. An expression vector harboring a two target gene-specific gRNA has been constructed based on the published system of Xing et al. (2014). Reverse and forward primers for two FH1 gene-specific gRNAs (**Supplementary Data S1**) were designed using the CRISPR-P web tool (Lei et al., 2014). Target sequence fragments were amplified from pCBC-DT1T2 using Q5 polymerase (New England Biolabs) according to the manufacturer's instructions and cloned into the vector pHSE401E using the Golden Gate cloning reaction with enzymes from New England Biolabs. The resulting construct expressing two gRNAs was transformed into *A. thaliana* Col-8 by standard *Agrobacterium tumefaciens* infiltration. Transgenic plants were selected by hygromycin resistance and presence of homozygous single base insertion was verified by sequencing (for primer see **Supplementary Data S1**, for *fh1*:CRISPR allele sequence see **Supplementary Data S2**).

Crosses between *fh1-1, fh1-4*, and *arpc5-1* mutants and between actin nucleator mutants and reporter lines were performed to obtain double mutants and to introduce cytoskeletal markers into mutant backgrounds. For detection of cytoskeletal organization and dynamics, fluorescent marker protein constructs GFP–MAP4 and UBQ : Lifeact-GFP were used as described previously (Cvrčková and Oulehlová, 2017). In the indicated experiments, lines carrying the GFP-FABD (Voigt et al., 2005) or GFP-TUA6 (Ueda et al., 1999) markers were employed. Additional plant lines expressing these proteins were obtained by crossing or by transformation in case of UBQ : Lifeact-GFP, which was introduced into the double mutant *fh1arpc5* using the floral dip method. A transformed plant with a moderate level of fluorescence was subsequently backcrossed with wt and wt and single or double mutant lines were selected out of its progeny.

### Growth Conditions

For morphological analyses of pavement cells and cytoskeleton observations, plants were grown *in vitro* at 22°C with a 16 hour-light/8 hour-dark cycle on vertical MS plates following stratification of imbibed seed by 2 days at 4°C, as described previously (Rosero et al., 2016). Plants for propagation, crossing, macroscopical observations, and trichome morphology analysis were grown in peat pellets (Jiffy) at 22°C with a 16 hour-light/8 hour-dark cycle.

### Organ Area Measurements

Cotyledon or leaf area was measured on images captured by a Nikon D 3200 camera using the ImageJ software (Schindelin et al., 2015). Approximately 20 cotyledons and leaves per genotype were analyzed.

### Pavement Cell Morphometric Analysis and Quantification of Epidermal Gaps

Pavement cell shape parameters were determined from the adaxial epidermis of the apical third of cotyledons or leaves stained with 1 μM FM4-64 for 1–2 hours in the dark (Rosero et al., 2016). Images were taken using confocal laser-scanning microscope Zeiss TCS 880 with a 20 x water-immersion objective in ZEISS ZEN Black program.

Cell shape parameters of area, circularity, solidity, and aspect ratio were determined using the built-in circularity function of ImageJ by measuring all in-focus cells crossing or touching the diagonal of a microscopic field as described previously (Oulehlová et al., 2019). This will be further referred to as the semi-manual method. For an independent estimate of area, circularity and solidity, as well as for determination of pavement cell lobe parameters (lobe number and average equatorial lobe width), the PaCeQuant software (Möller et al., 2017; Möller et al., 2019) involving automated algorithmic cell segmentation was employed. Comparison of cell area, circularity, and solidity values obtained by both approaches from the same images revealed a non-linear relationship between results of the two methods, with PaCeQuant often reporting smaller cell size and higher circularity and solidity than the semi-manual approach (**Supplementary Data S3**). Based on visual examination of the progress of cell detection in PaCeQuant, we believe that the reason may be less efficient segmentation of large or very lobed cells by the PaCeQuant algorithm compared to small or moderately lobed ones. To avoid this source of bias and maintain compatibility with our previous studies, we subsequently used the semi-manual approach for estimation of cell shape parameters while PaCeQuant was applied to determine the lobe parameters. The only exception was acquisition of data for principal component analysis (PCA) where PaCeQuant was employed for collecting both cell and lobe parameters to ensure match between values originating from the same individual cells.

Gaps between the pavements cells were counted from the same images as described previously (Sahi et al., 2018).

All measurements were done in 2–3 biological replicates, each including at least 60, but usually more than 100, cells per genotype from at least 7, but usually over 20 plants.

### Cytoskeleton Structure and Dynamics Evaluation

Stills of the actin and microtubule networks in epidermal pavement cells were taken by a Zeiss LSM880 microscope with a Plan-Apochromat 40 x/1.2 W objective and 488-nm argon laser for excitation, videos were taken by an inverted spinning disc confocal microscope (Yokogawa CSU-X1 on a Nikon Ti-E platform, laser box Agilent MLC400, camera Andor Ixon) with 100 x/1,45 O plan apochromatic objective, excitation laser line set at 488 nm, and image interval 1 s as described previously (Rosero et al., 2016). Cytoskeleton bundling and density were quantified as described previously (Higaki et al., 2010) with minor modifications (Rosero et al., 2013; Rosero et al., 2019).

Cytoskeletal dynamics measurements were done in two biological replicates: with at least 40 cells from at least 20 videos analyzed using the QuACK method (Cvrčková and Oulehlová, 2017).

Actin networks in developing trichromes were visualized using an inverted spinning disc confocal microscope (Zeiss Axio Observer 7 microscope with a vertical stage equipped with a Yokogawa CSU-W1 spinning disk unit and Photometrics Prime 95B camera) with Zeiss Plan Apochromat 40 x/1.2 W objective and 488-nm laser excitation line.

### Trichome Shape Analysis

First true leaves of 24 days after germination (DAG) plants were cleared for 6 days in a solution prepared by mixing 120 g chloral hydrate, 7.5 ml glycerol, and 150 ml of water, embedded in the same solution and imaged using the transmission light microscope Olympus Provis AX 70 with a 20 x water-immersion objective. Trichome branch number was counted visually and branch length was measured after manual tracing on photos using the length measurement tool of ImageJ. All measurements were done in two biological replicates involving analysis of all trichomes from at least five longitudinal leaf halves originating from different plants (with a minimum of 100 trichomes per genotype). Measurements for terminal branch length determination were performed in at least 50 three-branched trichomes, and at least 15 (but usually more than 20) four-branched trichomes per genotype. For PCA of 3-branched trichome shape, at least 50 trichomes were measured.

### Statistics and Data Presentation

Unless stated otherwise, statistical evaluation of quantitative data was performed using ANOVA with post-hoc Tukey HSD test using the online calculator available at http://astatsa.com/. Results of cytoskeletal dynamics measurements, which could not be assumed to fulfill the normal distribution requirement for ANOVA, were statistically evaluated using the Kruskal–Wallis test followed by pairwise Wilcoxon–Mann–Whitney test with Bonferroni correction as described previously (Cvrčková and Oulehlová, 2017). The significance of between-genotype differences in trichome branch number distribution (categorical data) was assessed using pairwise Chi-square test with Yates correction for low count categories and Benjamini-Hochberg correction for multiplicity. PCA was performed using the PAST (PAleontological STatistics) software (Hammer et al., 2001) version 3.25 or 3.26. Box plots were generated using BoxPlotR (Spitzer et al., 2014).

## Results

### Plants Lacking Both *FH1* and *ARPC5* Show No Obvious Phenotypic Defects

To examine effects of simultaneous perturbation of both actin nucleation systems, we generated mutant plants doubly homozygous for *fh1* and *arpc5* by crossing single mutants. While our analyses were restricted to a single, well-characterized, loss of function allele of *arpc5* in the Columbia 0 background, which exhibited hallmark characteristics of ARP2/3 complex disruption in several previous studies (Li et al., 2003; Djakovic et al., 2006; Li et al., 2013; Sahi et al., 2018), we included three different *fh1* mutant alleles into our analyses. Besides of two T-DNA insertion mutants, *fh1-1* and *fh1-4,* derived from the Columbia 0 (Col-0) background (Rosero et al., 2013; Rosero et al., 2016; Oulehlová et al., 2019), we also generated a Columbia 8 (Col-8)-derived plant line incapable to express the FH1 protein using the CRISPR method, further referred to as *fh1:CRISPR*, and used it for some experiments.

**Figure 1:**
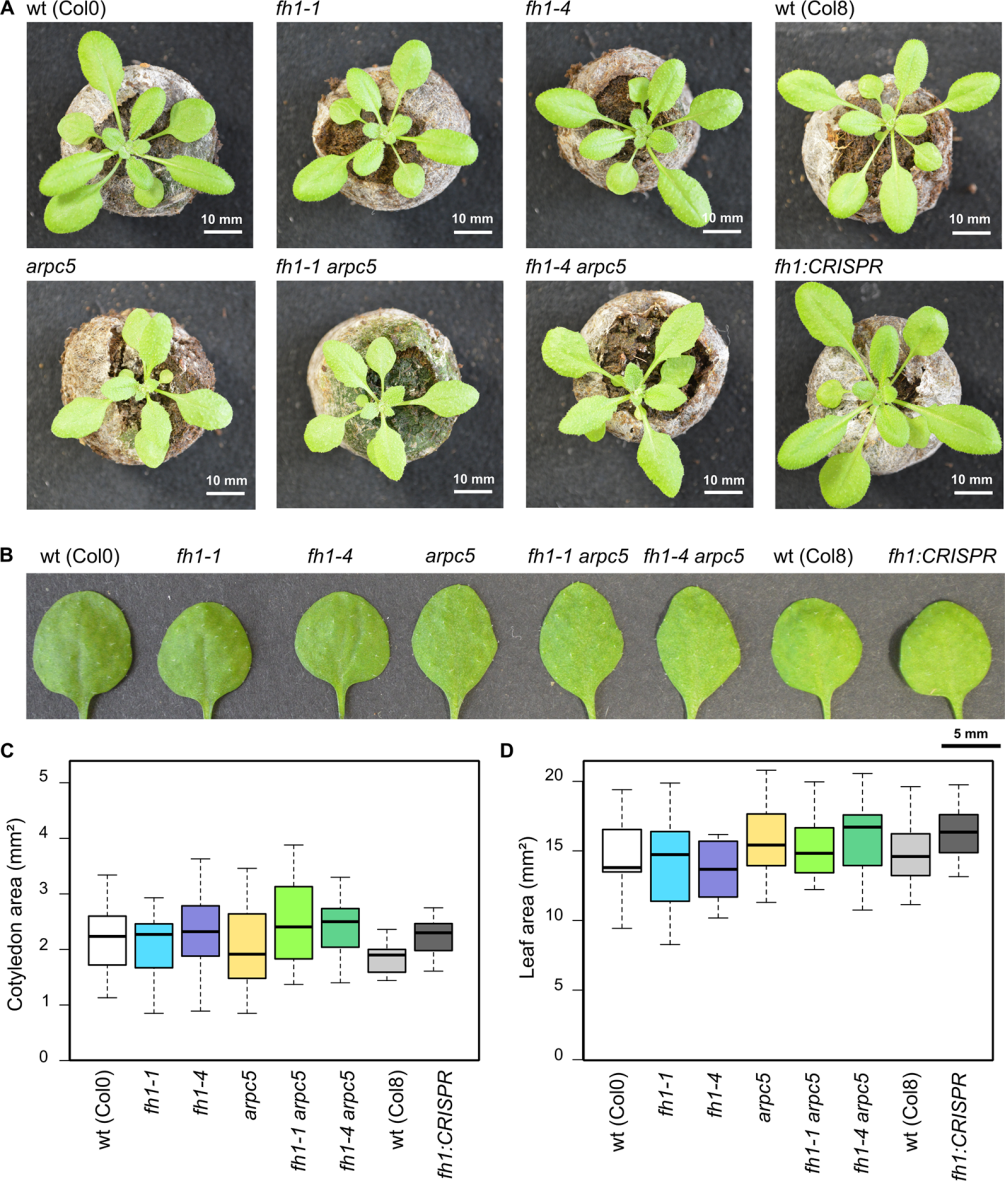
Actin nucleator mutants do not exhibit gross phenotypic alterations. **(A)** Representative photos of 21 DAG plants carrying the *arpc5* and *fh1* mutations analyzed in this report, double mutant plants, and relevant wild types (wt). **(B)** Representative photos of 1^st^ true leaves of 21 days after germination (DAG) plants. **(C)** Area of wt and mutant 5 DAG cotyledons. **(D)** Area of 1^st^ true leaves of 14 DAG wt and mutant plants. None of the between-genotype differences in **(C, D)** is statistically significant.

Given the relatively minor phenotypic consequences of both *arpc5* and *fh1* mutations, as well as the probable redundancy within the plant Class I formin family, we expected that the double mutants should be free from major developmental defects. Indeed, all single mutants examined, as well as double *fh1-1 arpc5-1*, and *fh1-4 arpc5-1* mutants, were viable, fertile, and indistinguishable from isogenic wt plants at the first macroscopic glance (**Figure 1A**). Upon closer inspection, however, a subtle leaf shape alteration was apparent in all plant lines involving *arpc5* (**Figure 1B**), albeit there was no significant difference in the size of either cotyledons (**Figure 1C**) or first true leaves (**Figure 1D**).

### *ARPC5* Is Epistatic Over *FH1* in Determining Cotyledon Pavement Cell Shape

**Figure 2:**
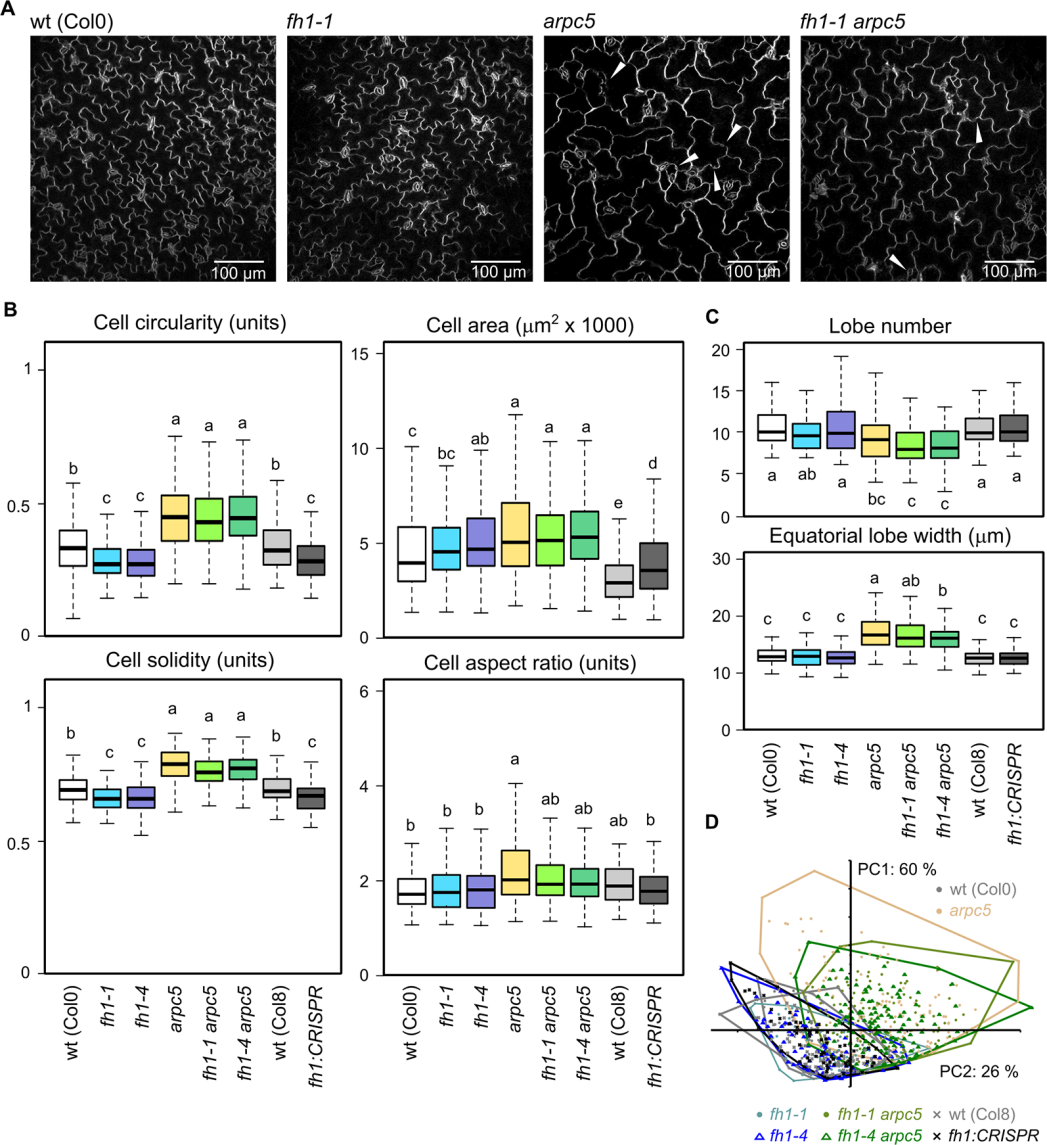
Pavement cell shape in the adaxial epidermis of 5 days after germination (DAG) cotyledons of actin nucleator mutants and wt plants. **(A)** Representative images of wt, *fh1-1, arpc5-1*, and *fh1-1 arpc5-1* cotyledon epidermis. Epidermal continuity defects are marked by arrowheads. **(B)** Selected morphometric parameters of cotyledon pavement cells as determined by the semi-manual approach (see *Methods*): cell circularity and solidity as measures of cell shape complexity, cell area, aspect ratio as a measure of shape anisotropy. **(C)** Average cell lobe number and equatorial lobe width as determined by PaCeQuant. **(D)** Scatter plot of PCA results for five morphometric parameters (cell circularity, solidity and area, lobe number, and average equatorial lobe width) determined by PaCeQuant, shown at an arbitrary scale. Values labeled by the same letters in **(B, C)** do not differ significantly (p *<* 0.05).

One of the hallmark phenotypic effects of mutations affecting the ARP2/3 complex subunits is reduced cotyledon pavement cell lobing (Mathur et al., 2003b; Li et al., 2003), while loss of FH1 has an opposite effect, i.e. increases pavement cells lobing in the cotyledon epidermis (Rosero et al., 2016; Oulehlová et al., 2019). These observations from single mutants have been confirmed in epidermal pavement cells of five DAG cotyledons, while pavement cell shape (as well as the quantitative parameters of circularity, solidity, and lobe width) of double *arpc5 fh1* mutants resembled those of single *arpc5* mutants (**Figures 2A–C**). The allelic status of *ARPC5* rather than *FH1* thus determines cotyledon pavement cell shape in the double mutants. Therefore, the *arpc5* mutation appears to be epistatic over *fh1* in the sense of the original Bateson's definition of epistasis (Phillips, 2008) as far as cotyledon pavement cell shape is concerned. Minor and only in some cases statistically significant differences consistent with this interpretation were observed also for two additional cell shape parameters—aspect ratio (**Figure 2B**) and lobe number (**Figure 2C**), indicating that single and double mutants involving *arpc5* have somewhat less elongated cells with fewer lobes.

In line with previous reports (Zhang et al., 2008; Rosero et al., 2016; Sahi et al., 2018) single *fh1* or *arpc5* mutations also brought about an increase in pavement cell size. The extent of this effect was comparable for both single mutants and for the double *fh1 arpc5* mutants, i.e. the effect of both mutations was not additive, nor did they compensate for each other's effects (**Figures 2 A, B**). Since no changes in organ size were observed (compare **Figures 1C, D**), mutant cotyledons must consist of fewer cells than wt ones, suggesting developmental coupling between cell division and cell expansion (compare Rosero et al., 2016).

PCA involving five parameters (cell area, circularity, solidity, lobe number, and lobe width) demonstrated that while cotyledon pavement cells of *fh1* mutants generally resemble those of wt plants, those of double *fh1 arpc5* mutants are clearly similar to those from single *arpc5* mutants (**Figure 2D**). Remarkably, PCA failed to visualize differences between wt and *fh1* plants in spite of the observed increase in cell lobing. We believe that this may be due to the method employed to collect data for this approach, which appears to suffer from a detection bias against highly lobed cells (see Methods and **Supplementary Data S3**).

The changes in cotyledon pavement cell shape and size observed in five DAG plants persisted at least until 14 DAG (**Supplementary Data S4**), confirming earlier observations (Zhang et al., 2008; Rosero et al., 2016; Sahi et al., 2018).

### Effects of *arpc5* and *fh1* Mutations on Cytoskeletal Organization and Dynamics

To investigate microfilament and microtubule organization and dynamics, we introduced fluorescent protein markers (GFP-MAP4 for microtubules and UBQ : Lifeact-GFP for microfilaments) previously used to investigate cytoskeletal dynamics in *fh1* plants (Rosero et al., 2016; Cvrčková and Oulehlová, 2017) also into *arpc5,* and *fh1 arpc5* double mutants. To further examine robustness and biological relevance of any cytoskeletal structure or dynamics alterations observed in the *arpc5* mutants, we prepared additional plant lines expressing alternative cytoskeletal markers in a genetic background impaired in the function of the ARP2/3 complex. These included *arpc5* and *arp2* plants carrying the microtubule marker GFP-TUA6 (Ueda et al., 1999), as well as lines carrying the actin reporter GFP-FABD, used in our previous work (Rosero et al., 2016), in the *arpc5*, *arp2*, or *arpc4* background.

**Figure 3:**
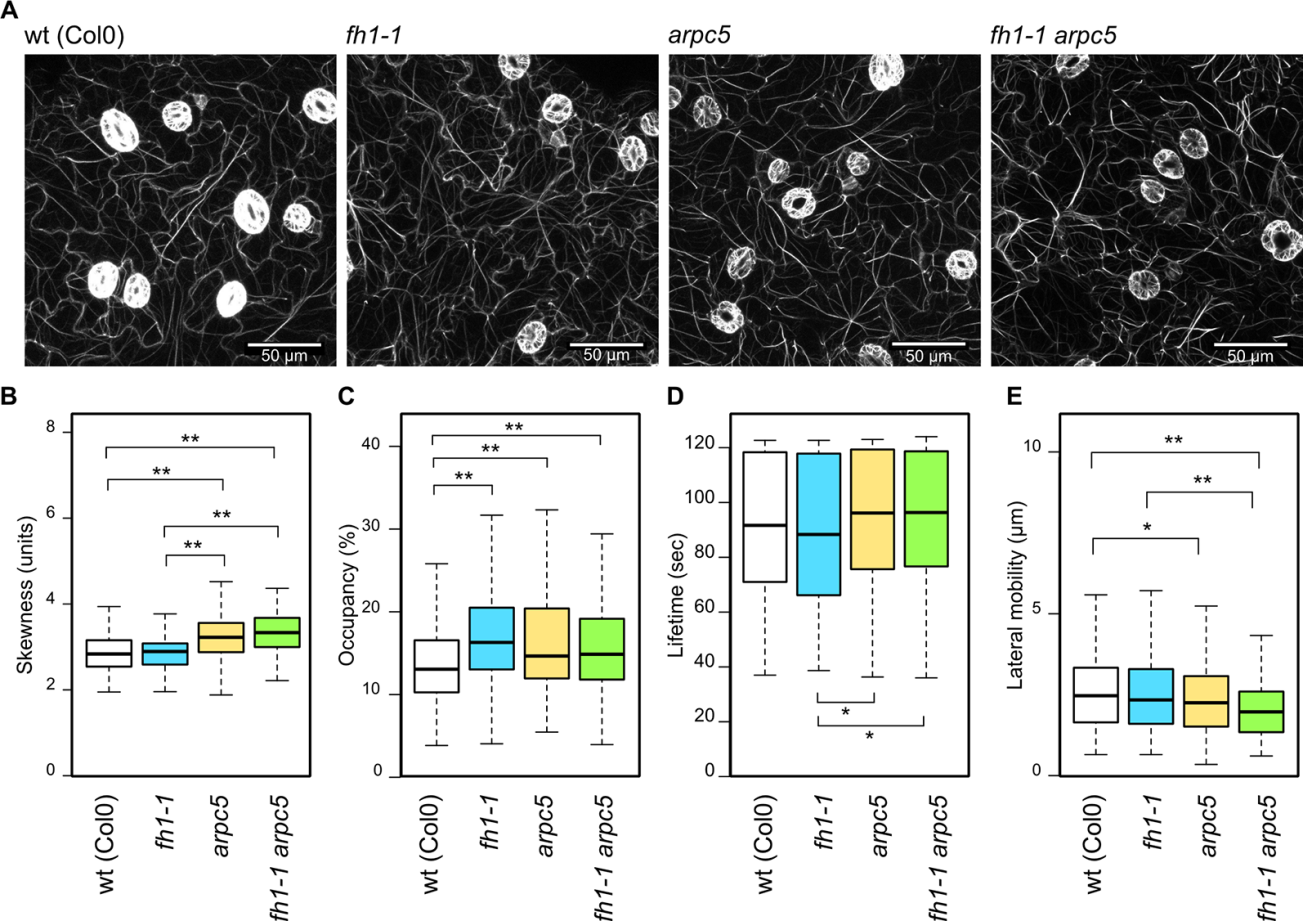
Actin cytoskeleton organization and dynamics in the adaxial cotyledon epidermis of 5 days after germination (DAG) actin nucleator mutants expressing Lifeact-GFP. **(A)** Representative images of microfilament organization in wt, *fh1-1, arpc5-1,* and *fh1-1 arpc5-1* seedlings. **(B)** Quantitative estimate of actin bundling, measured as skewness of fluorescence intensity distribution among labeled structures. **(C)** Quantitative estimate of actin network density, measured as pixel occupancy of skeletonized meshwork. **(D)** Microfilament structure lifetime, represented by maximum values observed over 120 s in structures crossing a 20 µm transect. **(E)** Microfilament structure lateral mobility, represented by maximum trajectories observed over 120 s in structures crossing a 20 µm transect. Statistical significance of differences is denoted by asterisks (* for *p <* 0.05, ** for *p <* 0.01).

Somewhat surprisingly, given the expected disruption of actin nucleation, no dramatic changes in actin organization were observed in any of the mutants examined (**Figure 3A**, **Supplementary Data S5**). Contrary to our previous observations in plants carrying the GFP-FABD actin marker, which suggested increased bundling and decreased density of microfilaments in *fh1* mutants compared to the wt (Rosero et al., 2013; Rosero et al., 2016), LifeAct-GFP-labeled microfilaments in cotyledon pavement cells of 5 DAG *fh1* mutants were bundled comparably (**Figure 3B**) but slightly denser (**Figure 3C**) than those in wt plants. In the *arpc5* mutants, as well as in double *fh1 arpc5* mutants, enhanced microfilament bundling was detected using the Lifeact-GFP marker (**Figure 3B**), together with a minor increase in actin filament density comparable to that seen in *fh1* mutants (**Figure 3C**). Increased actin bundling in *arpc5* plants was also confirmed using the alternative marker GFP-FABD, although no effect on microfilament density was observed; no significant change was, however, seen in *arp2* and *arpc4* mutants (**Supplementary Data S5**), previously shown to affect pavement cell shape in a manner similar to *arpc5* (Sahi et al., 2018).

While there was no significant influence of single actin nucleator mutations on the actin structures lifetime compared to the wt, *arpc5* and double *fh1 arpc5* mutants exhibited somewhat longer lifetimes than *fh1* mutants (**Figure 3D**). A trend toward decreased lateral actin mobility was observed in *fh1* mutants, although less dramatic than in our previous studies (Rosero et al., 2016; Cvrčková and Oulehlová, 2017). Remarkably, *arpc5* and *fh1 arpc5* double mutants exhibited a statistically significant effect in the same direction, namely decrease of lateral actin mobility (**Figure 3E**). While the biological relevance of these effects on microfilament dynamics remains questionable due to their low extent, as well as to the fact that no significant changes were found using the GFP-FABD marker in *arpc5*, *arp2*, or *arpc4* mutants (**Supplementary Data S5**), they fit the pattern already observed in pavement cell shape and actin organization—namely epistasis of *arpc5* over *fh1*. However, an additive effect of both mutations cannot be excluded, since the double *fh1 arpc5* mutant presents an even more pronounced decrease in actin lateral movement compared to wt and to *fh1* mutants (although the difference is not statistically significant).

**Figure 4:**
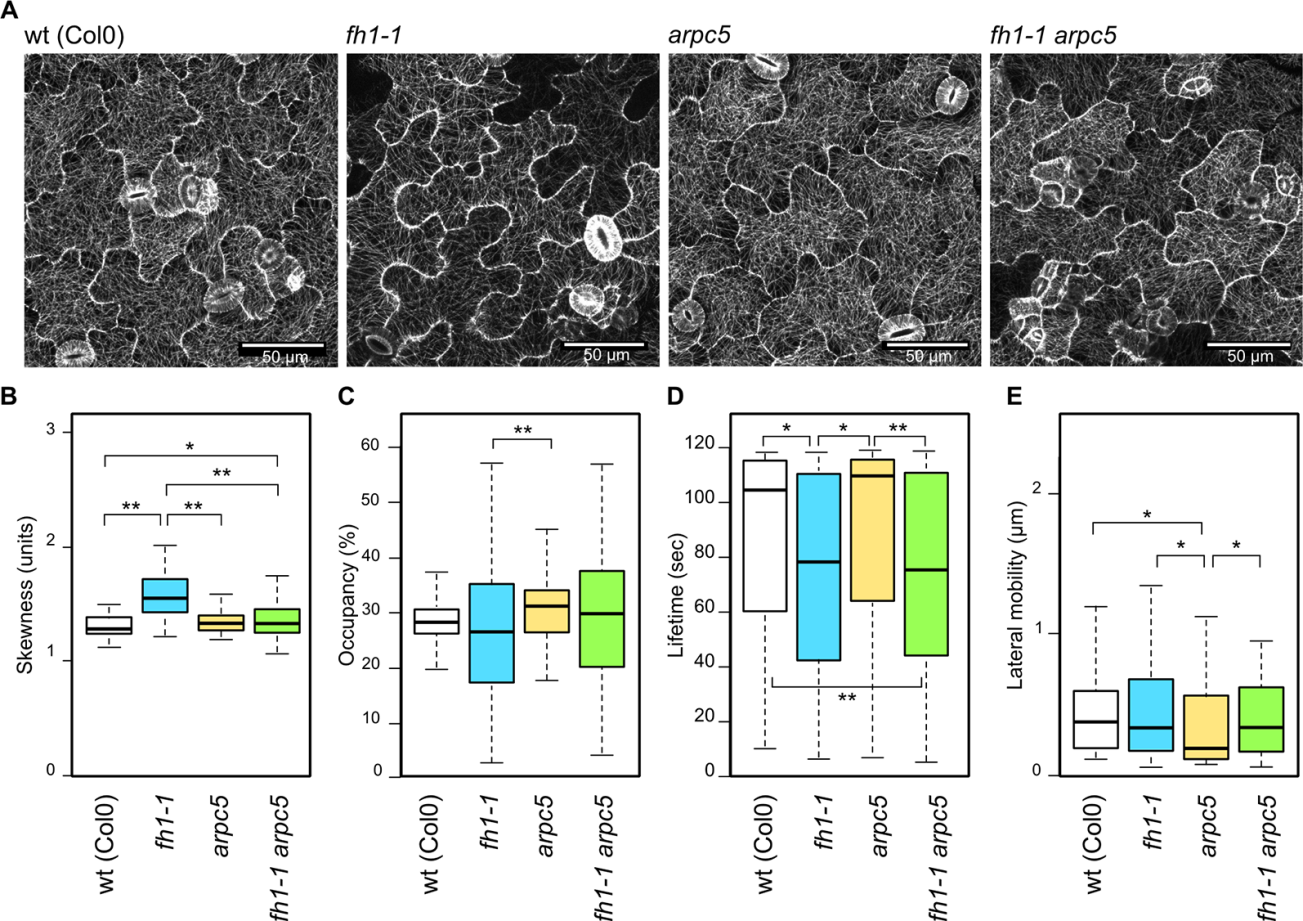
Microtubule organization and dynamics in the adaxial cotyledon epidermis of 5 days after germination (DAG) actin nucleator mutants expressing GFP-MAP4. **(A)** Representative images of microtubule organization in wt, *fh1-1, arpc5-1,* and *fh1-1 arpc5-1* seedlings. **(B)** Quantitative estimate of microtubule bundling, measured as skewness of fluorescence intensity distribution among labeled structures. **(C)** Quantitative estimate of microtubule network density, measured as pixel occupancy of skeletonized meshwork. **(D)** Microtubule or bundle lifetime, represented by maximum values observed over 120 s in structures crossing a 2 µm transect. **(E)** Microtubule or bundle lateral mobility, represented by maximum trajectories observed over 120 s in structures crossing a 2 µm transect. Statistical significance of differences is denoted by asterisks (* for *p <* 0.05, ** for *p <* 0.01).

Effects of actin nucleator mutations on microtubule arrangement, as documented using the GFP-MAP4 marker, are rather subtle (**Figure 4A**). The *arpc5* mutation did not affect microtubule organization but an increase in microtubule bundling was found in *fh1* mutants compared to wt, *arpc5,* and *fh1 arpc5* plants, consistent with epistasis of *arpc5* over *fh1* (**Figure 4B**). Minor differences were also found in the density of microtubule structures (**Figure 4C**). However, the alternative microtubule marker GFP-TUA6 did reveal some decrease in microtubule bundling in both *arpc5* and *arp2* mutants, though the extent of the effect was rather small (Supplementary Data Figure S6).Consistent with previous observations suggesting that the *fh1* mutant phenotype may be due mainly to altered microtubule function, though possibly secondary to changes in the actin cytoskeleton (Rosero et al., 2013; Rosero et al., 2016), pronounced changes in microtubule dynamics have been found in plants carrying the *fh1* mutation. These mutants exhibited a markedly shortened microtubule lifetime, while no change in the lifetime was found in *arpc5* mutants. Surprisingly, microtubule lifetime in double *fh1 arpc5* mutants was as short as in *fh1* mutants (**Figure 4D**), while only minor changes were detected in lateral microtubule mobility—namely a slight but significant decrease in *arpc5* mutants (**Figure 4E**). No changes in microtubule dynamics were observed in either *arpc5* or *arp2* mutants using the alternative microtubule marker GFP-TUA6 (**Supplementary Data S6**). Collectively, the microtubule dynamics parameters would thus suggest epistasis of *fh1* over *arpc5*, in contrast to the pattern observed in all other parameters analyzed so far.

### Mutations of Both Actin Nucleators Have Similar Effects on True Leaf Pavement Cell Shape

**Figure 5:**
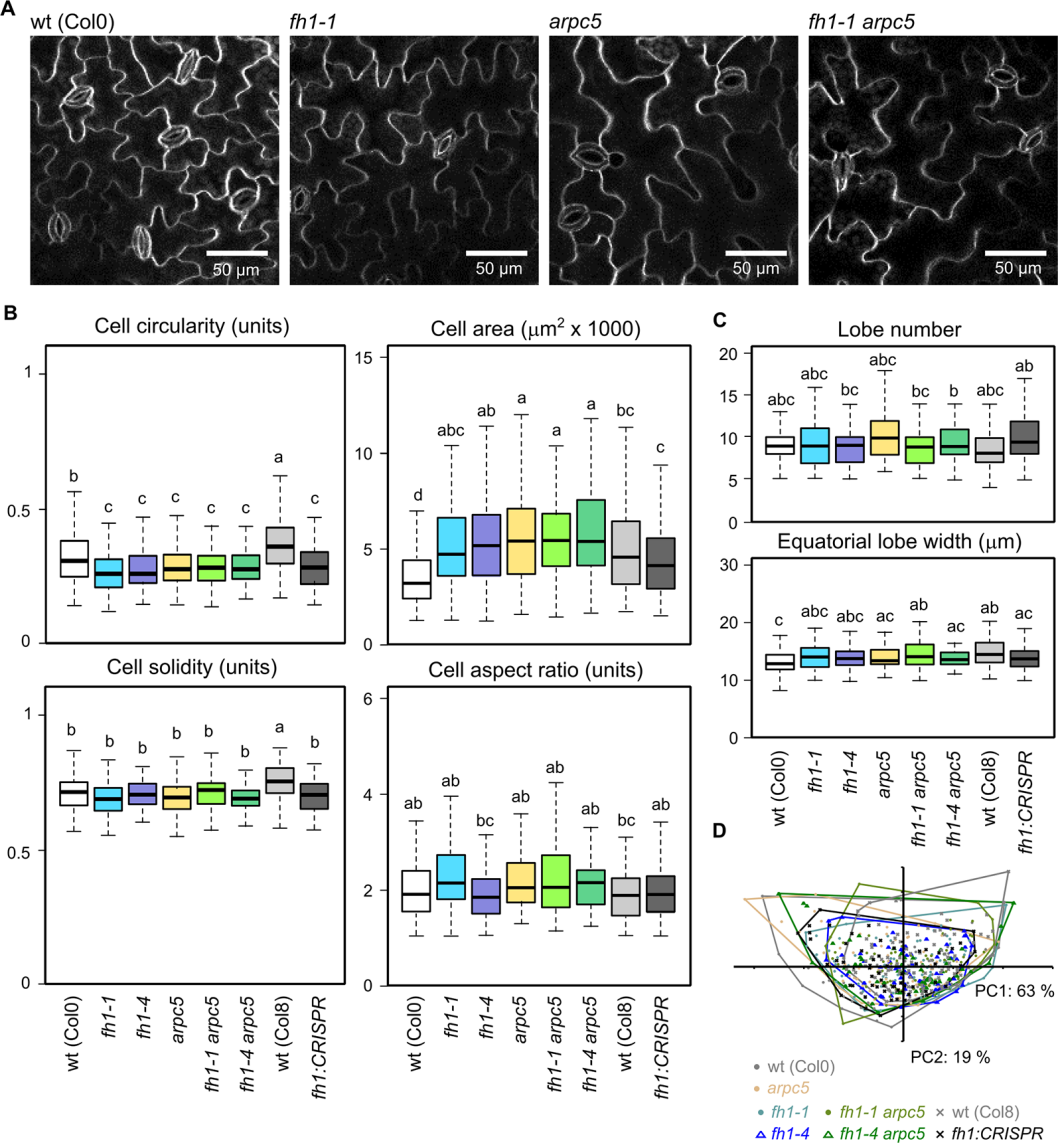
Pavement cell shape in the adaxial epidermis of 1^st^ true leaves of 14 days after germination (DAG) actin nucleator mutants and wt plants. **(A)** Representative images of wt, *fh1-1, arpc5-1,* and *fh1-1 arpc5-1* true leaf pavement cells. **(B)** Selected morphometric parameters of leaf epidermal pavement cells as determined by the semi-manual approach (see *Methods*): cell circularity and solidity as measures of cell shape complexity, cell area, aspect ratio as a measure of shape anisotropy. **(C)** Average cell lobe number and equatorial lobe width as determined by PaCeQuant. **(D)** Scatter plot of PCA results for five morphometric parameters (cell circularity, solidity and area, lobe number, and average equatorial lobe width) determined by PaCeQuant, shown at an arbitrary scale. Values labeled by the same letters in **(B, C)** do not differ significantly (p *<* 0.05).

We previously observed that the effect of the *arpc5* mutation on cotyledon pavement cell shape does not, somewhat surprisingly, extend to the true leaves. True leaf pavement cells of *arpc5* plants are larger than those of wt plants (similar to the cotyledon pavement cells), but they are more rather than less lobed, opposite to the situation in cotyledons (Sahi et al., 2018). We thus examined morphological parameters of true leaf pavement cells in our actin nucleator mutants (**Figure 5A**). The previously reported decrease in cell circularity in *arpc5* mutants has been reproduced, while *fh1* mutants still exhibited increased lobing, similar to the situation in cotyledons, and their cell circularity was thus indistinguishable from *arpc5* or the double mutants Single *fh1* or *arpc5* mutations and double *fh1 arpc5* mutation also caused similar increase in true leaf pavement cell size, except of the *fh1-CRISPR* allele that did not have significant effects (**Figure 5B**). Somewhat surprisingly, while the effects of the examined mutations on cell solidity mimicked those on circularity in cotyledon pavement cells, only minor changes in solidity and other parameters (aspect ratio, lobe number, or lobe width) were observed in true leaves (**Figures 5B, C**). Although leaf pavement cells in most mutants were larger than in wt plants (**Figure 5B**), no obvious changes in leaf size were observed, comparable to the situation in cotyledons (see **Figures 1A, B, D**).

Generally, the effect of *fh1* or *arpc5* mutations on epidermal cell shape in true leaves was much weaker than in the cotyledons. Overall leaf pavement cell shape similarity between all studied genotypes was also confirmed by PCA (**Figure 5D**).

### *ARPC5* is Epistatic Over *FH1* in Determining Epidermal Continuity

**Figure 6:**
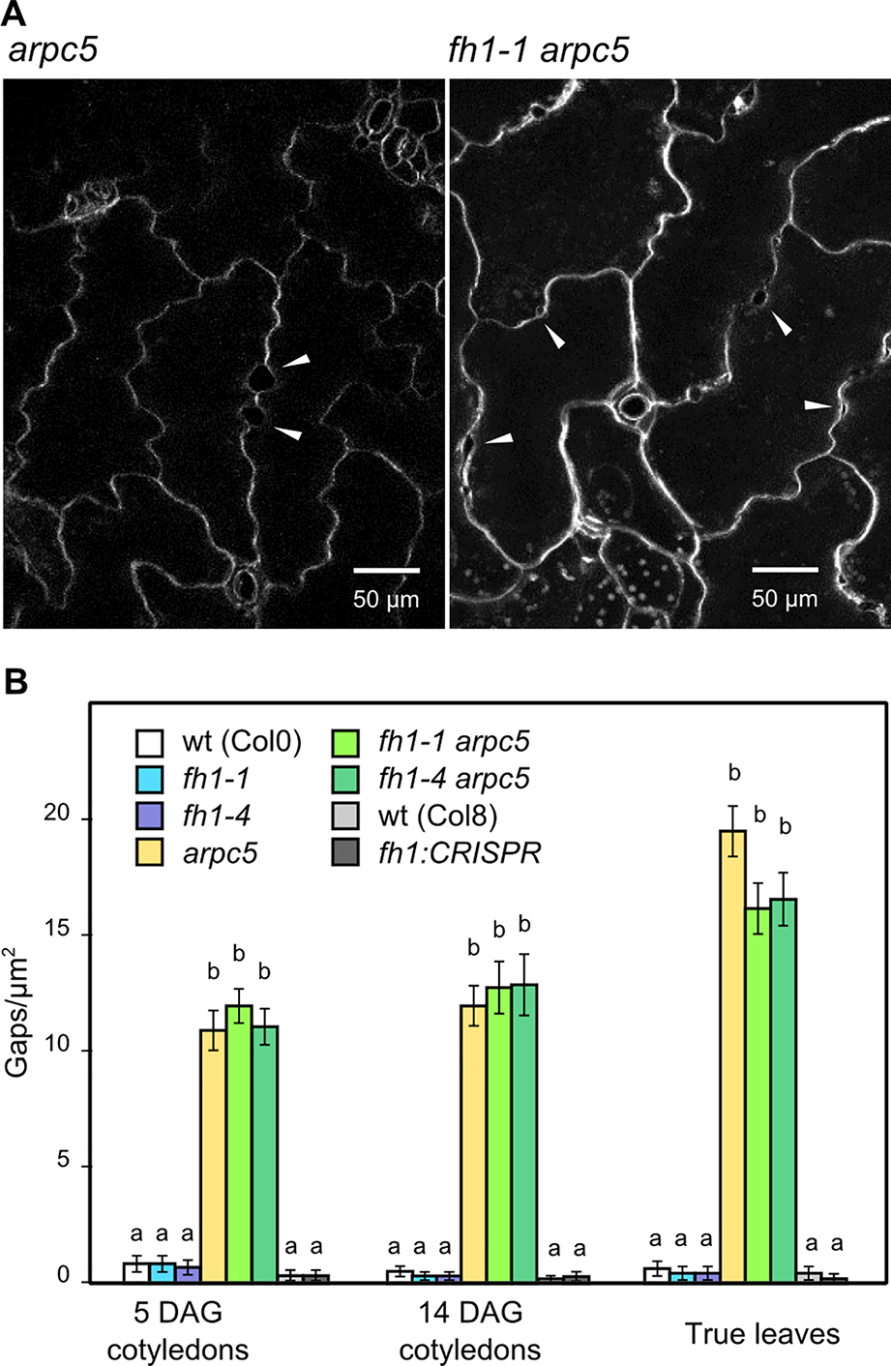
Epidermal continuity defects in actin nucleator mutants. **(A)** Close-up of 14 days after germination (DAG) cotyledon epidermis of *arpc5-1* and *fh1-1 arpc5-1* mutants, epidermal gaps marked by arrowheads. **(B)** Quantification of gap density in cotyledons and first true leaves. Values labeled by the same letters do not differ significantly (p < 0.05).

Mutations disrupting the ARP2/3 complex function often result in the formation of gaps between adjacent epidermal pavement cells (see e.g. Mathur et al., 2003a; Mathur et al., 2003b; Sahi et al., 2018). We also observed gaps between pavement cells in our plants carrying the *arpc5* mutation, regardless of the allelic status of *FH1* (see **Figures 2A** and **6A**). This prompted us to investigate epidermal gap formation quantitatively in younger and older cotyledons, as well as in true leaves. We found that epidermal gaps appear, almost exclusively, in both cotyledons and true leaves of single *arpc5* mutants, as well as in double *fh1 arpc5* mutants, while the *fh1* mutation neither causes epidermal continuity defects nor affects the formation of epidermal gaps caused by *arpc5* (**Figure 6B**). Thus, *arpc5* mutation appears to be epistatic over *fh1* also with respect to its effect of epidermal continuity.

### Effects of *arpc5* and *fh1* Mutations on Trichome Shape

Mutations affecting the function of the ARP2/3 complex are known to cause characteristic deformations of the typical architecture of the branched unicellular *A. thaliana* trichomes. This is the case also of *arpc5*, whose trichomes are distorted and have shorter branches than those of wt plants (Mathur et al., 2003b; Li et al., 2003). On the other hand, trichomes of *fh1* mutants are, at the first glance, shaped normally but mutant plants exhibit a somewhat larger fraction of four-branched trichomes compared to the wt (Oulehlová et al., 2019). We did not observe any gross abnormalities in microfilament organization during early trichome development in single *arpc5* or *fh1* mutants (**Supplementary Data S7**), although possible differences in the extent of microfilament bundling may deserve further attention.

**Figure 7:**
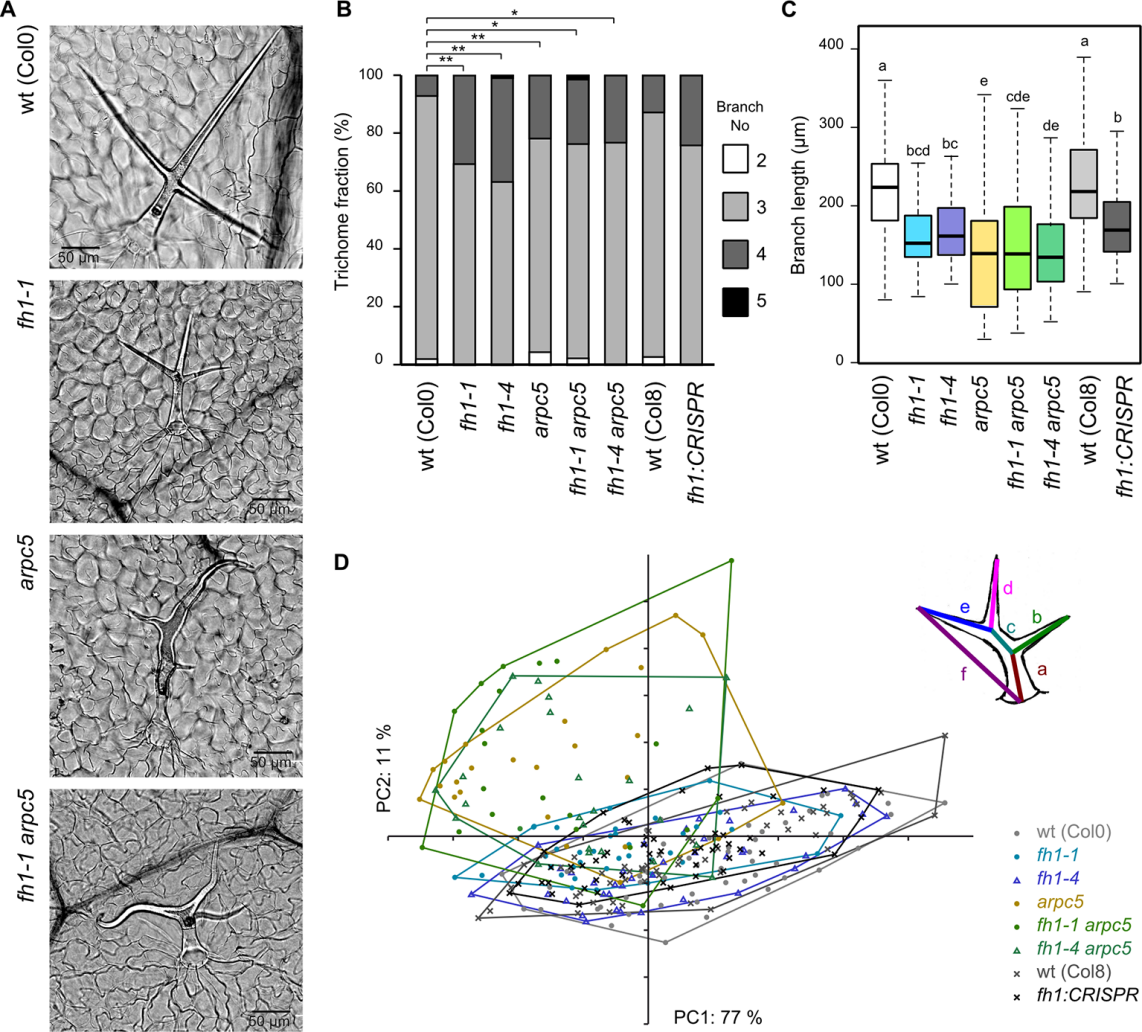
Trichome shape in wt plants and actin nucleator mutants. **(A)** Typical trichomes of wt, *fh1-1, arpc5-1*, and *fh1-1 arpc5-1* plants. **(B)** Distribution of trichome branch numbers. Statistical significance of between-genotype differences is denoted by asterisks (* for *p <* 0.05, **for *p <* 0.01). **(C)** Length of terminal branches in three-branched trichomes. Values labeled by the same letters do not differ significantly (p *<* 0.05). **(D)** Scatter plot of principal component analysis (PCA) results for six length parameters (a–f, see inset for definition) describing the three-branched trichome shape and size, shown at an arbitrary scale.

We confirmed these observations in single mutants and found that double *fh1 arpc5* mutants also have distorted trichomes (**Figure 7A**). An increase in the frequency of four-branched trichomes was observed not only in *fh1* plants, but also in *arpc5* mutants, as well as in double *fh1 arpc5* mutants, which did not significantly differ from *fh1* plants in this parameter (**Figure 7B**). Terminal branches of typical three-branched trichomes of *fh1* mutants were shorter than those of wt plants, and their length was further reduced in *arpc5* mutants. Trichomes of double mutants resembled those of *arpc5* plants also in this parameter (**Figure 7C**). A similar, though less distinct, difference in trichome branch length was documented also for four-branched trichomes (**Supplementary Data S8**). PCA involving six parameters (the length of each internal or apical branch and overall trichome size) confirmed that three-branched *fh1* trichomes resemble those of wt plants, while those of double *fh1 arpc5* mutants are indistinguishable from single *arpc5* mutants (**Figure 7D**).

Thus, the double *fh1 arpc5* mutants resemble single *arpc5* mutants with respect to trichome shape and are indistinguishable from either single mutant with respect to trichome branching. Together, these observations suggest epistasis of *arpc5* over *fh1* with respect to trichome development.

## Discussion

In contrast to opisthokonts, who possess multiple actin nucleation systems, plants currently seem to engage only two classes of actin nucleators: the ARP2/3 complex and formins (e.g. Blanchoin and Staiger, 2010; Vaškovičová et al., 2013; Yanagisawa et al., 2013). Multiple reports describe the role of one or another of these actin nucleation machineries in plant cell morphogenesis and development both in *Arabidopsis* (e.g. Li et al., 2003; Rosero et al., 2013; Rosero et al., 2016; Sahi et al., 2018) and in other angiosperms (e.g. Yang et al., 2011; Zhang Z. et al., 2011; Hossain et al., 2012; Huang et al., 2013; Facette et al., 2015; Gavrin et al., 2015; Qiu et al., 2015; Qi et al., 2017). In the present study, the impact of simultaneous perturbation of both actin nucleation systems on *Arabidopsis* epidermal cells shaping was investigated for the first time, with focus on interdigitated epidermal pavement cells and trichomes.

Actin nucleation may be expected to be essential for eukaryotic cell functioning. Indeed, in budding yeast (Winter et al., 1999) or fission yeast (e.g. Cabrera et al., 2011), loss of some ARP2/3 complex subunits is lethal; mutations affecting other subunits cause severe cell morphogenesis defects. Disrupting the metazoan ARP2/3 complex also tends to result in tissue- or organ-level problems rather than cellular lethality (e.g. Di Nardo et al., 2005; van der Kammen et al., 2017). In plants, the ARP2/3 complex appears to be needed for proper cell morphogenesis and development but not for cell viability. Perturbation of the ARP2/3 complex in the moss *Physcomitrella patens* prevents completion of the developmental cycle and disturbs polar growth and cell morphogenesis (Harries et al., 2005; Finka et al., 2008). In angiosperms, including *A. thaliana*, loss of the ARP2/3 complex or its regulators results in altered shape of trichomes and epidermal pavement cells, but does not cause gross developmental defects (Mathur et al., 2003a; see Ivakov and Persson, 2013; Facette et al., 2015). Impairment of ARP2/3 function resulted in additional rather subtle phenotypic changes—for example, altered stomata closure dynamics in several mutants affecting ARP2/3 complex function, including the *arpc5-1* mutant analyzed in the present study (Li et al., 2013). A possible reason for the lack of dramatic phenotypic effects may be that plant cells can generate new actin filaments by other mechanisms, such as nucleation by formins or elongation of short actin filaments generated by severing proteins (Michelot et al., 2007; Staiger et al., 2009). ARP2/3 complex subunits are encoded by single copy or duplicated genes in *Arabidopsis* (Li et al., 2003), and the knock-out of individual single-copy genes leads to similar phenotypic effects (Li et al., 2003; Mathur et al., 2003b; Djakovic et al., 2006; Li et al., 2013; Sahi et al., 2018) underscoring their functionality within the same complex.

Unlike ARP2/3 complex subunits, formins comprise large gene families in most lineages, including metazoans and plants (Grunt et al., 2008). Thus, loss of a single formin may be compensated by its functionally overlapping relatives. *Arabidopsis* plants lacking the main housekeeping Class I formin FH1 are viable, fertile, and free from major developmental defects but exhibit altered root growth, cytoskeletal drug sensitivity, cytoskeletal dynamics, and also changed pavement cell shape. Remarkably, these phenotypic effects of the *fh1* mutation, as well as its impact on cytoskeletal dynamics, were largely phenocopied in seedlings treated by SMIFH2, a specific inhibitor of formin activity (Rosero et al., 2013; Rosero et al., 2016). A T-DNA insertion disrupting the closest FH1 paralog, FH2 (At2g43800), did not significantly alter pavement cell shape, although it slightly increased pavement cell size (Rosero, 2013). Since pavement cell size appears to be easily influenced by numerous conditions, including, e.g., expression of cytoskeletal markers (Rosero et al., 2016), we consider these observations consistent with a prominent role of FH1 in vegetative development at least at the seedling stage, although contribution of other formins (including, but not limited to, FH2) would clearly deserve further attention.

Here we show that also double *fh1 arpc5* mutants exhibit no major growth or developmental defects possibly again due to functional redundancy of the numerous formin paralogs. This enabled us to investigate the mutual relationships of the two actin nucleation systems in cytoskeletal organization and epidermal cell morphogenesis.

Plant cell shape is determined by a complex interplay between vacuole-driven turgor pressure, cell wall synthesis and remodeling, mechanical stress, cytoskeletal dynamics, and membrane trafficking (Szymanski and Cosgrove, 2009; Sampathkumar et al., 2014; Belteton et al., 2018). Epidermal pavement cells and branched unicellular *Arabidopsis* trichomes have long served as models to study these processes (see, e.g. Ivakov and Persson, 2013; Sapala et al., 2018; Altartouri et al., 2019). Microtubules are believed to contribute significantly to the control of pavement cells lobes initiation (Panteris and Galatis, 2005; Armour et al., 2015; Belteton et al., 2018), whereas orchestrated action of both actin and microtubule cytoskeleton is needed for later and final stages of pavement cell development (Smith and Oppenheimer, 2005; Zhang C. et al., 2011). Changes in cytoskeletal organization responsible for the layout of pavement cell lobes, especially microtubule bundling, occur very early in development, when the cells are still convex (Armour et al., 2015). In plants carrying the actin marker GFP-FABD whose expression itself causes changes in pavement cell size and shape, no differences were observed between cells that ceased expressing the transgene before day 4 after germination and those with continuous expression (Rosero et al., 2016), indicating participation of the actin cytoskeleton in very early steps of pavement cell shape determination. *Arabidopsis* mutants with impaired actin nucleation commonly exhibit altered pavement cell shape and size (e.g. Li et al., 2003; Mathur et al., 2003a; Mathur et al., 2003b; El-Assal et al., 2004; Basu et al., 2004; Basu et al., 2005; Rosero et al., 2016; Sahi et al., 2018; Oulehlová et al., 2019), again consistent with microfilaments contributing to early microtubule rearrangements that establishes the lobe layout (compare Cvrčková et al., 2016), albeit cytoskeletal organization and dynamics during early pavement cell development in these mutants remains to be characterized.

Cotyledon pavement cells of *arpc5* plants are less lobed than in wt plants (Mathur et al., 2003b; Li et al., 2003), while *fh1* mutants show an opposite phenotype, i.e. increased cotyledon pavement cell shape complexity (Rosero et al., 2016; Oulehlová et al., 2019). Here we show that cotyledon pavement cells of double *fh1 arpc5* mutants resemble those of *arpc5* plants. The *arpc5* mutation is thus epistatic over *fh1* with respect to cotyledon pavement cell shape determination. However, in true leaves the *arpc5* mutant exhibits increased rather than decreased pavement cell shape complexity (Sahi et al., 2018), and all mutants examined in this study, i.e. *arpc5*, *fh1*, and *fh1 arpc5*, displayed a similar decrease in true leaf epidermal pavement cell circularity and an increase in cell size compared to wt plants. Thus, pavement cell shaping in cotyledons (embryonic leaves) and true post-embryonic leaves apparently engages the actin nucleation mechanisms in a different manner. This is not surprising, because post-germination cotyledon growth relies solely on cell expansion in the absence of cell division, while true leaf cells simultaneously divide and expand (Tsukaya et al., 1994). Additional mechanisms such as, e.g., mechanical stress and tension may contribute to cell shaping differently in cotyledons versus true leaves. Although epidermis growth properties may influence organ shape (Savaldi-Goldstein et al., 2007), in our system pavement cells shape and size did not influence organ size, consistent with previous observations that whole organ size is barely, if at all, affected by cell size and shape changes induced by actin nucleator disruption, indicating efficient compensation for altered cell division or cell expansion at the organ development level (e.g. Rosero et al., 2016; Sahi et al., 2018).

Another classic model cell type used to study cell shaping regulated by orchestrated action of actin and microtubules are leaf trichomes. Trichome branches initiation appears to be controlled mainly by microtubules (Sambade et al., 2014) and trichome branches growth by ARP2/3-nucleated microfilaments (Mathur et al., 1999; Szymanski et al., 1999; Le et al., 2003; Schwab et al., 2003; Li et al., 2003; El-Assal et al., 2004; Basu et al., 2004; Basu et al., 2005; Yanagisawa et al., 2015; Tian et al., 2015). Remarkably, components of the ARP2/3 complex, as well as the FH1 protein, localize to nascent tips during early trichome development (Yanagisawa et al., 2015; Yanagisawa et al., 2018; Oulehlová et al., 2019). Along with the well-known “distorted trichome” phenotype (Mathur et al., 2003b), *arpc5* plants exhibited shorter terminal trichome branches and an increase in trichome branch number. Also *fh1* mutants show increased trichome branch number (Oulehlová et al., 2019). Trichome branch initiation can be induced by transient microtubule stabilization in branching-deficient mutants (Mathur and Chua, 2000) or by microtubule-stabilizing mutation (Buschmann et al., 2009). However, *fh1* mutants have more dynamic microtubules in epidermal pavement cells (Rosero et al., 2016; Cvrčková and Oulehlová, 2017; see also below). Unless the effects of the *fh1* mutation differ between trichomes and pavement cells, a possible explanation of the apparent paradox is that increased microtubule mobility in *fh1* mutants may promote generation of transient microtubular structures required for branch initiation, while microtubule stabilization increases the lifetime of such structures. This would be consistent with our hypothesis explaining increased pavement cell lobing in *fh1* mutants by enhanced generation of microtubule bundles at future pavement cell lobe necks (Cvrčková et al., 2016). Additional observations, focusing on cytoskeletal dynamics during early stages of trichome ontogeny, are needed to test this hypothesis. The distorted trichome phenotype of double *fh1 arpc5* mutants indicates epistasis of *arpc5* over *fh1*, as in the case of cotyledon pavement cells, and is consistent with cell wall expansion required for proper branch elongation being controlled by ARP2/3 (Yanagisawa et al., 2015; Yanagisawa et al., 2018).

To gain insight into the cytoskeletal basis of observed epidermal cell shape differences, we analyzed organization and dynamics of microfilaments and microtubules in epidermal pavement cells of single and double mutants. Changes were expected in both cytoskeletal systems, since some ARP2/3 complex subunits (Zhang et al., 2013b; Havelková et al., 2015) and at least some formins (Deeks et al., 2010; Li et al., 2010; Yang et al., 2011; Wang et al., 2012; Wang et al., 2013), bind to microtubules in addition to their actin-related functions. Thus, phenotypic effects of ARP2/3 complex or formin perturbation may be mediated by either actin or microtubules, or by both cytoskeletal systems.

In general, loss of FH1 affected the microtubular network more dramatically than the microfilaments. Enhanced microtubule dynamics in *fh1* mutants was observed, consistent with our previous observations (Rosero et al., 2016; Cvrčková and Oulehlová, 2017). Disruption of ARP2/3 complex function has led to some noticeable, though rather weak, effects upon the actin cytoskeleton that might secondarily affect microtubules *via* actin-microtubule interactions. However, the biological relevance of these effects is questionable because they were only partly reproduced using an alternative actin marker. In the double *fh1 arpc5* mutants, microfilament organization and dynamics generally resembled that in single *arpc5* mutants, unlike the microtubule behavior, which reflected that observed in *fh1* mutants.

In contrast to our previous observations (Rosero et al., 2016) we did not observe a significant increase in actin bundling in the *fh1* mutants compared to wt plants. We attribute this discrepancy to the nature and expression level of the markers used to label the actin cytoskeleton in our previous study, where we employed GFP-FABD and Lifeact-mRFP markers expressed under the strong 35S promoter. Those marker lines exhibited transgene silencing in subsequent generations, leading to decreased and patchy marker expression after crossing into T-DNA insertional lines, which possibly resulted in the loss of ability to detect fine, non-bundled microfilaments (Rosero et al., 2016). Moreover, while GFP-FABD detects a less bundled microfilament network, it rather dramatically reverses the pavement cell shape alteration brought about by *fh1* mutations. This effect, not observed with a Lifeact-based marker, makes interpretation of results obtained using GFP-FABD in formin mutants problematic (Rosero et al., 2016). Although Lifeact marker constructs stabilize actin network when strongly expressed, moderate expression levels enable detection of fine, highly dynamic microfilaments (van der Honing et al., 2011; Dyachok et al., 2014; Kijima et al., 2018). In the present study, a pUBQ-driven |Lifeact-GFP actin marker, which was not silenced, was used. Although microfilament bundling was still observed, we believe that our current measurements provide a more realistic estimate of mutant actin dynamics than those in our previous report where the preferential detection of bundles was further aggravated by silencing (Rosero et al., 2016).

At the first glance, the observed effects of actin nucleator impairment on the cytoskeletal organization and dynamics were not mirrored in cell shape properties in the double *fh1 arpc5* mutants. Microtubules, which are essential for pavement cell lobe and trichome branch initiation, are more dynamic in *fh1* mutants and thus prone to re-organize more readily into structures that establish the layout of a complex cell shape (Rosero et al., 2016; Cvrčková et al., 2016). In the double mutants the microtubule cytoskeleton is as dynamic as in single *fh1* mutants but resulting cell shapes are less complex, resembling instead single *arpc5* mutants. This could be due to ARP2/3-dependent nucleation of new actin being required to progress from lobe or trichome branch initiation (“layout establishment”) to actual lobe or branch expansion that determines the final cell shape complexity (Panteris and Galatis, 2005; Yanagisawa et al., 2015). Thus, the loss of dynamic actin in ARP2/3-defective mutants seems to play a dominant role in adopting the final shape.

Cell expansion is intimately linked to cell wall polymer deposition (Altartouri et al., 2019). Indeed, qualitative changes in cell wall composition were detected at later stages of ARP2/3-defective mutant development (Sahi et al., 2018). Consistent with previous reports describing formation of epidermal gaps in such mutants (e.g. Mathur et al., 2003a; Mathur et al., 2003b; Sahi et al., 2018), cell adhesion defects were observed in the epidermis of *arpc5* mutant seedlings, regardless of their *fh1* state. Such defects are also common in plants with altered cell wall carbohydrate composition, especially altered pectin content (Bouton et al., 2002) and pectin deficiency-induced signaling from the cell wall to cell wall synthesis machinery (Verger et al., 2016). Our observations thus are consistent with the ARP2/3-controlled actin filaments or the ARP2/3 complex itself being involved in cell wall biosynthesis by a yet unknown mechanism.

In summary, our results imply that the formin FH1 and the ARP2/3 complex share complementary roles in some aspects of cotyledon pavement cell morphogenesis while they act synergistically in the shaping of trichomes and true leaf pavement cells. This suggests that the control of actin dynamics, affecting also microtubule organization and dynamics, is developmentally regulated, and that the importance of any particular actin nucleation mechanism changes in the course of ontogeny.

## Author's Note

Simultaneous impairment of the ARP2/3 complex and the main housekeeping formin FH1 activity has been employed to characterize the contribution of these two actin-nucleating systems to epidermal cells morphogenesis, revealing their distinct roles in pavement cell and trichome shaping.

## Data Availability Statement

All data supporting the conclusions of this article are included in the article/**Supplementary Material**.

## Author Contributions

VŽ formulated the initial working hypothesis. VŽ, KS and FC conceived and designed the research. PC, DO, AR and EK constructed the plant lines. PC, EK and AR performed initial phenotypic characterization of mutants. DO, JM, PC, AR, KS and EK performed microscopic observations. PC, EK and FC performed image analysis and statistical evaluation of results. PC, KS and FC drafted the manuscript. PC and FC prepared the figures. FC, VŽ and KS performed final manuscript editing.

## Funding

Research at the Department of Experimental Plant Biology has been supported by the Grant Agency of the Czech Republic (projects 15-02610S and 19-10845S); work performed at the Institute of Experimental Botany was supported by the Ministry of Education, Youth and Sports of the Czech Republic from European Regional Development Fund Project “Centre for Experimental Plant Biology” No. CZ.02.1.01/0.0/0.0/16_019/0000738. The microscopy facilities used in this work were supported by the European Regional Development Fund and the state budget of the Czech Republic (projects CZ.1.05/4.1.00/16.0347 and CZ.2.16/3.1.00/21515), by Ministry of Education, Youth and Sports of the Czech Republic (project LM2015062 Czech-BioImaging), and by the European Research Council Grant No. 803048.

## Conflict of Interest

The authors declare that the research was conducted in the absence of any commercial or financial relationships that could be construed as a potential conflict of interest.

## References

[B1] Altartouri B. Bidhendi A. J. Tani T. Suzuki J. Conrad C. Chebli Y. (2019). Pectin chemistry and cellulose crystallinity govern pavement cell morphogenesis in a multi-step mechanism. Plant Physiol. 181, 127–141. 10.1104/pp.19.00303 31363005 PMC6716242

[B2] Armour W. J. Barton D. A. Law A. M. K. Overall R. L. (2015). Differential growth in periclinal and anticlinal walls during lobe formation in Arabidopsis cotyledon pavement cells. Plant Cell 27, 2484–2500. 10.1105/tpc.114.126664 26296967 PMC4815096

[B3] Bartolini F. Gundersen G. G. (2010). Formins and microtubules. Biochim. Biophys. Acta 1803, 164–173. 10.1016/j.bbamcr.2009.07.006 19631698 PMC2856479

[B4] Bashline L. Lei L. Li S. Gu Y. (2014). Cell wall, cytoskeleton, and cell expansion in higher plants. Mol. Plant 7, 586–600. 10.1093/mp/ssu018 24557922

[B5] Basu D. El-Assal S. E. D. Le J. Mallery E. L. Szymanski D. B. (2004). Interchangeable functions of Arabidopsis PIROGI and the human WAVE complex subunit SRA1 during leaf epidermal development. Development 131, 4345–4355. 10.1242/dev.01307 15294869

[B6] Basu D. Le J. El-Assal S. E. D. Huang S. Zhang C. H. Mallery E. L. (2005). DISTORTED3/SCAR2 is a putative Arabidopsis WAVE complex subunit that activates the Arp2/3 complex and is required for epidermal morphogenesis. Plant Cell 17, 502–524. 10.1105/tpc.104.027987 15659634 PMC548822

[B7] Belteton S. A. Sawchuk M. G. Donohoe B. S. Scarpella E. Szymanski D. B. (2018). Reassessing the roles of PIN proteins and anticlinal microtubules during pavement cell morphogenesis. Plant Physiol. 176, 432–449. 10.1104/pp.17.01554 29192026 PMC5761804

[B8] Blanchoin L. Staiger C. J. (2010). Plant formins: diverse isoforms and unique molecular mechanism. Biochim. Biophys. Acta 1803, 201–206. 10.1016/j.bbamcr.2008.09.015 18977251

[B9] Blanchoin L. Boujemaa-Paterski R. Henty J. L. Khurana P. Staiger C. J. (2010). Actin dynamics in plant cells: a team effort from multiple proteins orchestrates this very fast-paced game. Curr. Opin. Plant Biol. 13, 714–723. 10.1016/j.pbi.2010.09.013 20970372

[B10] Bouton S. Leboeuf E. Mouille G. Leydecker M. T. Talbotec J. Granier F. (2002). QUASIMODO1 encodes a putative membrane-bound glycosyltransferase required for normal pectin synthesis and cell adhesion in Arabidopsis. Plant Cell 14, 2577–2590. 10.1105/tpc.004259 12368506 PMC151237

[B11] Buschmann H. Hauptmann M. Niessing D. Lloyd C. W. Schäffner A. R. (2009). Helical growth of the Arabidopsis mutant *tortifolia2* does not depend on cell division patterns but involves handed twisting of isolated cells. Plant Cell 21, 2090–2106. 10.1105/tpc.108.061242 19638477 PMC2729594

[B12] Cabrera R. Suo J. Young E. Chang E. C. (2011). *Schizosaccharomyces pombe* Arc3 is a conserved subunit of the Arp2/3 complex required for polarity, actin organization, and endocytosis. Yeast 28, 495–503. 10.1002/yea.1853 21449051 PMC3107344

[B13] Campellone K. G. Webb N. J. Znameroski E. A. Welch M. D. (2008). WHAMM is an Arp2/3 complex activator that binds microtubules and functions in ER to Golgi transport. Cell 134, 148–161. 10.1016/j.cell.2008.05.032 18614018 PMC2556884

[B14] Cao L. Henty-Ridilla J. L. Blanchoin L. Staiger C. J. (2016). Profilin-dependent nucleation and assembly of actin filaments controls cell elongation in Arabidopsis. Plant Physiol. 170, 220–233. 10.1104/pp.15.01321 26574597 PMC4704583

[B15] Carlier M. F. Shekhar S. (2017). Global treadmilling coordinates actin turnover and controls the size of actin networks. Nat. Rev. Mol. Cell Biol. 18, 389–401. 10.1038/nrm.(2016)172 28248322

[B16] Carnahan R. H. Gould K. L. (2003). The PCH family protein, Cdc15p, recruits two F-actin nucleation pathways to coordinate cytokinetic actin ring formation in *Schizosaccharomyces pombe* . J. Cell Biol. 162, 851–862. 10.1083/jcb.200305012 12939254 PMC2172828

[B17] Copeland S. J. Thurston S. F. Copeland J. W. (2016). Actin- and microtubule-dependent regulation of Golgi morphology by FHDC1. Mol. Biol. Cell 27, 260–276. 10.1091/mbc.e15-02-0070 26564798 PMC4713130

[B18] Cvrčková F. Oulehlová D. (2017). A new kymogram-based method reveals unexpected effects of marker protein expression and spatial anisotropy of cytoskeletal dynamics in plant cell cortex. Plant Methods 13, 19. 10.1186/s13007-017-0171-9 28360928 PMC5368923

[B19] Cvrčková F. Oulehlová D. Žárský V. (2014). Formins: linking cytoskeleton and endomembranes in plant cells. Int. J. Mol. Sci. 16, 1–18. 10.3390/ijms16010001 25546384 PMC4307232

[B20] Cvrčková F. Oulehlová D. Žárský V. (2016). On growth and formins. Plant Signal. Behav. 11, e1155017. 10.1080/15592324.2016.1155017 26910482 PMC4883901

[B21] Cvrčková F. (2013). Formins and membranes: anchoring cortical actin to the cell wall and beyond. Front. Plant Sci. 4, 436. 10.3389/fpls.2013.00436 24204371 PMC3817587

[B22] Deeks M. J. Fendrych M. Smertenko A. Bell K. S. Oparka K. Cvrčková F. (2010). The plant formin AtFH4 interacts with both actin and microtubules, and contains a newly identified microtubule-binding domain. J. Cell Sci. 123, 1209–1215. 10.1242/jcs.065557 20332108

[B23] Di Nardo A. Cicchetti G. Falet H. Hartwig J. H. Stossel T. P. Kwiatkowski D. J. (2005). Arp2/3 complex-deficient mouse fibroblasts are viable and have normal leading-edge actin structure and function. Proc. Natl. Acad. Sci. U.S.A. 102, 16263–16268. 10.1073/pnas.0508228102 16254049 PMC1283463

[B24] Diao M. Ren S. Wang Q. Qian L. Shen J. Liu Y. (2018). Arabidopsis formin 2 regulates cell-to-cell trafficking by capping and stabilizing actin filaments at plasmodesmata. eLfe 7, e36316. 10.7554/eLife.36316 PMC6126924 30113309

[B25] Djakovic S. Dyachok J. Burke M. Frank M. J. Smith L. G. (2006). BRICK1/HSPC300 functions with SCAR and the ARP2/3 complex to regulate epidermal cell shape in Arabidopsis. Development 133, 1091–1100. 10.1242/dev.02280 16481352

[B26] Dominguez R. (2016). The WH2 domain and actin nucleation: necessary but insufficient. Trends Biochem. Sci. 41, 478–490. 10.1016/j.tibs.2016.03.004 27068179 PMC4884163

[B27] Dyachok J. Sparks J. A. Liao F. Wang Y. S. Blancaflor E. B. (2014). Fluorescent protein-based reporters of the actin cytoskeleton in living plant cells: fluorophore variant, actin binding domain, and promoter considerations. Cytoskeleton 71, 311–327. 10.1002/cm.21174 24659536

[B28] Edwards M. Zwolak A. Schafer D. A. Sept D. Dominguez R. Cooper J. A. (2014). Capping protein regulators fine-tune actin assembly dynamics. Nat. Rev. Mol. Cell Biol. 15, 677–689. 10.1038/nrm3869 25207437 PMC4271544

[B29] El-Assal S. E. D. Le J. Basu D. Mallery E. L. Szymanski D. B. (2004). Arabidopsis GNARLED encodes a NAP125 homolog that positively regulates ARP2/3. Curr. Biol. 14, 1405–1409. 10.1016/j.cub.2004.06.062 15296760

[B30] Facette M. R. Park Y. Sutimantanapi D. Luo A. Cartwright H. N. Yang B. (2015). The SCAR/WAVE complex polarizes PAN receptors and promotes division asymmetry in maize. Nat. Plants 1, 14024. 10.1038/nplants.2014.24 27246760

[B31] Fišerová J. Schwarzerová K. Petrášek J. Opatrný Z. (2006). ARP2 and ARP3 are localized to sites of actin filament nucleation in tobacco BY-2 cells. Protoplasma 227, 119–128. 10.1007/s00709-006-0146-6 16736254

[B32] Finka A. Saidi Y. Goloubinoff P. Neuhaus J. M. Zryd J. P. Schaefer D. G. (2008). The knock-out of ARP3a gene affects F-actin cytoskeleton organization altering cellular tip growth, morphology and development in moss *Physcomitrella patens* . Cytoskeleton 65, 769–784. 10.1002/cm.20298 18613119

[B33] Fu Y. Li H. Yang Z. B. (2002). The ROP2 GTPase controls the formation of cortical fine F-actin and the early phase of directional cell expansion during Arabidopsis organogenesis. Plant Cell 14, 777–794. 10.1105/tpc.001537 11971134 PMC150681

[B34] Gavrin A. Jansen V. Ivanov S. Bisseling T. Fedorova E. (2015). ARP2/3-mediated actin nucleation associated with symbiosome membrane is essential for the development of symbiosomes in infected cells of *Medicago truncatula* root nodules. Mol. Plant-Microbe Int. 28, 605–614. 10.1094/MPMI-12-14-0402-R 25608180

[B35] Grikscheit K. Grosse R. (2016). Formins at the junction. Trends Biochem. Sci. 41, 148–159. 10.1016/j.tibs.2015.12.002 26732401

[B36] Grunt M. Žárský V. Cvrčková F. (2008). Roots of angiosperm formins: the evolutionary history of plant FH2 domain-containing proteins. BMC Evol. Biol. 8, 115. 10.1186/1471-2148-8-115 18430232 PMC2386819

[B37] Gurel P. S. Hatch A. L. Higgs H. N. (2014). Connecting the cytoskeleton to the endoplasmic reticulum and Golgi. Curr. Biol. 24, R660–R672. 10.1016/j.cub.2014.05.033 25050967 PMC4174561

[B38] Hammer Ø. Harper D. A. T. Ryan P. D. (2001). PAST: Paleontological statistics software package for education and data analysis. Palaeontol. Electron. 4, 1–9.

[B39] Harries P. A. Pan A. Quatrano R. S. (2005). Actin-related protein2/3 complex component ARPC1 is required for proper cell morphogenesis and polarized cell growth in *Physcomitrella patens* . Plant Cell 17, 2327–2339. 10.1105/tpc.105.033266 16006580 PMC1182492

[B40] Havelková L. Nanda G. Martinek J. Bellinvia E. Sikorová L. Šlajcherová K. (2015). Arp2/3 complex subunit ARPC2 binds to microtubules. Plant Sci. 241, 96–108. 10.1016/j.plantsci.2015.10.001 26706062

[B41] Henty-Ridilla J. L. Rankova A. Eskin J. A. Kenny K. Goode B. L. (2016). Accelerated actin filament polymerization from microtubule plus ends. Science 352, 1004–1009. 10.1126/science.aaf1709 27199431 PMC5179141

[B42] Higaki T. Kutsuna N. Sano T. Kondo N. Hasezawa S. (2010). Quantification and cluster analysis of actin cytoskeletal structures in plant cells: role of actin bundling in stomatal movement during diurnal cycles in Arabidopsis guard cells. Plant J. 61, 156–165. 10.1111/j.1365-313x.2009.04032.x 20092030

[B43] Hossain M. D. Liao J. James E. K. Sato S. Tabata S. Jurkiewicz A. (2012). *Lotus japonicus* ARPC1 is required for rhizobial infection. Plant Physiol. 160, 917–928. 10.1104/pp.112.202572 22864583 PMC3461565

[B44] Huang J. Kim C. M. Xuan Y. Liu J. Kim T. H. Kim B. K. (2013). Formin homology 1 (OsFH1) regulates root-hair elongation in rice (*Oryza sativa*). Planta 237, 1227–1239. 10.1007/s00425-013-1838-8 23334469

[B45] Hubert T. Perdu S. Vandekerckhove J. Gettemans J. (2011). γ-Tubulin localizes at actin-based membrane protrusions and inhibits formation of stress-fibers. Biochem. Biophys. Res. Commun. 408, 248–252. 10.1016/j.bbrc.2011.04.007 21473851

[B46] Ivakov A. Persson S. (2013). Plant cell shape: modulators and measurements. Front. Plant Sci. 4, 439. 10.3389/fpls.2013.00439 24312104 PMC3832843

[B47] Jimenez-Lopez J. C. Wang X. Kotchoni S. O. Huang S. Szymanski D. B. Staiger J. C. (2014). Heterodimeric capping protein from Arabidopsis is a membrane-associated, actin-binding protein. Plant Physiol. 166, 1312–1328. 10.1104/pp.114.242487 25201878 PMC4226361

[B48] Kijima S. T. Staiger C. J. Katoh K. Nagasaki A. Ito K. Uyeda T. Q. P. (2018). Arabidopsis vegetative actin isoforms, AtACT2 and AtACT7, generate distinct filament arrays in living plant cells. Sci. Rep. 8, 4381. 10.1038/s41598-018-22707-w 29531328 PMC5847576

[B49] Le J. El Assal S. E. D. Basu D. Saad M. E. Szymanski D. B. (2003). Requirements for Arabidopsis ATARP2 and ATARP3 during epidermal development. Curr. Biol. 13, 1341–1347. 10.1016/S0960-9822(03)00493-7 12906796

[B50] Lei Y. Lu L. Liu H. Y. Li S. Xing F. Chen L. L. (2014). CRISPR-P: A web tool for synthetic single-guide RNA design of CRISPR-system in plants. Mol. Plant 7, 1494–1496. 10.1093/mp/ssu044 24719468

[B51] Li S. D. Blanchoin L. Yang Z. B. Lord E. M. (2003). The putative Arabidopsis Arp2/3 complex controls leaf cell morphogenesis. Plant Physiol. 132, 2034–2044. 10.1104/pp.103.028563 12913159 PMC181288

[B52] Li Y. Shen Y. Cai C. Zhong C. Zhu L. Yuan M. (2010). The type II Arabidopsis formin14 interacts with microtubules and microfilaments to regulate cell division. Plant Cell 22, 2710–2726. 10.1105/tpc.110.075507 20709814 PMC2947165

[B53] Li X. Li J. H. Wang W. Chen N. Z. Ma T. S. (2013). ARP2/3 complex-mediated actin dynamics is required for hydrogen peroxide-induced stomatal closure in Arabidopsis. Plant Cell Environ. 37, 1547–1560. 10.1111/pce.12259 24372484

[B54] Li J. Staiger B. H. Henty-Ridilla J. L. Abu-Abied M. Sadot E. Blanchoin L. (2014). The availability of filament ends modulates actin stochastic dynamics in live plant cells. Mol. Biol. Cell 25, 1263–1275. 10.1091/mbc.e13-07-0378 24523291 PMC3982992

[B55] Liu R. Abreu-Blanco M. T. Barry K. C. Linardopoulou E. V. Osborn G. E. Parkhurst S. M. (2009). Wash functions downstream of Rho and links linear and branched actin nucleation factors. Development 136, 2849–2860. 10.1242/dev.035246 19633175 PMC2730411

[B56] Martinière A. Gayral P. Hawes C. Runions J. (2011). Building bridges: formin1 of Arabidopsis forms a connection between the cell wall and the actin cytoskeleton. Plant J. 66, 354–365. 10.1111/j.1365-313X.2011.04497.x 21241388

[B57] Mathur J. Chua N. H. (2000). Microtubule stabilization leads to growth reorientation in Arabidopsis trichomes. Plant Cell 12, 465–477. 10.1105/tpc.12.4.465 10760237 PMC139846

[B58] Mathur J. Spielhofer P. Kost B. Chua N. (1999). The actin cytoskeleton is required to elaborate and maintain spatial patterning during trichome cell morphogenesis in *Arabidopsis thaliana* . Development 126, 5559–5568. 10572033 10.1242/dev.126.24.5559

[B59] Mathur J. Mathur N. Kernebeck B. Hülskamp M. (2003a). Mutations in actin-related proteins 2 and 3 affect cell shape development in Arabidopsis. Plant Cell 15, 1632–1645. 10.1105/tpc.011676 12837952 PMC165406

[B60] Mathur J. Mathur N. Kirik V. Kernebeck B. Srinivas B. P. Hülskamp M. (2003b). Arabidopsis CROOKED encodes for the smallest subunit of the ARP2/3 complex and controls cell shape by region specific fine F-actin formation. Development 130, 3137–3146. 10.1242/dev.00549 12783786

[B61] Mathur J. (2005). The ARP2/3 complex: giving plant cells a leading edge. Bioessays 27, 377–387. 10.1002/bies.20206 15770684

[B62] Michelot A. Berro J. Guérin C. Boujemaa-Paterski R. Staiger C. J. Martiel J. L. (2007). Actin-filament stochastic dynamics mediated by ADF/Cofilin. Curr. Biol. 17, 825–833. 10.1016/j.cub.2007.04.037 17493813

[B63] Möller B. Poeschl Y. Plötner R. Bürstenbinder K. (2017). PaCeQuant: a tool for high-throughput quantification of pavement cell shape characteristics. Plant Physiol. 175, 998–1017. 10.1104/pp.17.00961 28931626 PMC5664455

[B64] Möller B. Poeschl Y. Klemm S. Bürstenbinder K. (2019). Morphological analysis of leaf epidermis pavement cells with PaCeQuant. Methods Mol. Biol. 1992, 329–349. 10.1007/978-1-4939-9469-4_22 31148049

[B65] Oulehlová D. Kollárová E. Cifrová P. Pejchar P. Žárský V. Cvrčková F. (2019). Arabidopsis class I formin FH1 relocates between membrane compartments during root cell ontogeny and associates with plasmodesmata. Plant Cell Physiol. 60, 1855–1870. 10.1093/pcp/pcz102 31135031

[B66] Panteris E. Galatis B. (2005). The morphogenesis of lobed plant cells in the mesophyll and epidermis: organization and distinct roles of cortical microtubules and actin filaments. New Phytol. 167, 721–732. 10.1111/j.1469-8137.2005.01464.x 16101909

[B67] Paredez A. R. Somerville C. R. Ehrhardt D. W. (2006). Visualization of cellulose synthase demonstrates functional association with microtubules. Science 312, 1491–1495. 10.1126/science.1126551 16627697

[B68] Peremyslov V. V. Cole R. A. Fowler J. E. Dolja V. V. (2015). Myosin-powered membrane compartment drives cytoplasmic streaming, cell expansion and plant development. PloS One 10, e0139331. 10.1371/journal.pone.0139331 26426395 PMC4591342

[B69] Phillips P. C. (2008). Epistasis – the essential role of gene interactions in the structure and evolution of genetic systems. Nat. Rev. Genet. 9, 855–867. 10.1038/nrg2452 18852697 PMC2689140

[B70] Pizarro-Cerdá J. Chorev D. S. Geiger B. Cossart P. (2017). The diverse family of Arp2/3 complexes. Trends Cell Biol. 27, 93–100. 10.1016/j.tcb.2016.08.001 27595492 PMC7098815

[B71] Pleskot R. Pejchar P. Žárský V. Staiger C. J. Potocký M. (2012). Structural insights into the inhibition of actin-capping protein by interactions with phosphatidic acid and phosphatidylinositol (4,5)-bisphosphate. PloS Comput. Biol. 8, e1002765. 10.1371/journal.pcbi.1002765 23133367 PMC3486809

[B72] Qi J. Greb T. (2017). Cell polarity in plants: the Yin and Yang of cellular functions. Curr. Opin. Plant Biol. 35, 105–110. 10.1016/j.pbi.2016.11.015 27918938 PMC7212042

[B73] Qi T. Wang J. Sun Q. Day B. Guo J. Ma Q. (2017). TaARPC3 contributes to wheat resistance against the stripe rust fungus. Front. Plant Sci. 8, 1245. 10.3389/fpls.2017.01245 28769954 PMC5513970

[B74] Qiu L. Lin J. Xu J. Sato S. Parniske M. Wang T. L. (2015). SCARN, a novel class of SCAR protein that is required for root hair infection during legume nodulation. PloS Genet. 11, e1005623. 10.1371/journal.pgen.1005623 26517270 PMC4627827

[B75] Rosero A. Žárský V. Cvrčková F. (2013). AtFH1 formin mutation affects actin filament and microtubule dynamics in *Arabidopsis thaliana* . J. Exp. Bot. 64, 585–597. 10.1093/jxb/ers351 23202131 PMC3542049

[B76] Rosero A. Oulehlová D. Stillerová L. Schiebertová P. Grunt M. Žárský V. (2016). Arabidopsis FH1 formin affects cotyledon pavement cell shape by modulating cytoskeleton dynamics. Plant Cell Physiol. 57, 488–504. 10.1093/pcp/pcv209 26738547

[B77] Rosero A. Oulehlová D. Žárský V. Cvrčková F. (2019). Visualizing and quantifying *in vivo* cortical cytoskeleton structure and dynamics. Meth. Mol. Biol. 1992, 135–149. 10.1007/978-1-4939-9469-4_9 31148036

[B78] Rosero A. (2013). Role of formins in the organization and dynamics of intracellular structures in Arabidopsis thaliana. [PhD thesis]. [Prague (CZ)]: Charles University Available at https://is.cuni.cz/webapps/zzp/detail/84878/(accessed December 28, 2019).

[B79] Rotty J. D. Wu C. Bear J. E. (2013). New insights into the regulation and cellular functions of the ARP2/3 complex. Nat. Rev. Mol. Cell Biol. 14, 7–12. 10.1038/nrm3492 23212475

[B80] Rotty J. D. Wu C. Haynes E. M. Suarez C. Winkelman J. D. Johnson H. E. (2015). Profilin-1 serves as a gatekeeper for actin assembly by Arp2/3-dependent and -independent pathways. Dev. Cell 32, 54–67. 10.1016/j.devcel.2014.10.026 25543281 PMC4296256

[B81] Saedler R. Mathur N. Srinivas B. P. Kernebeck B. Hülskamp M. Mathur J. (2004). Actin control over microtubules suggested by DISTORTED2 encoding the Arabidopsis ARPC2 subunit homolog. Plant. Cell Physiol. 45, 813–822. 10.1093/pcp/pch103 15295064

[B82] Sahi V. P. Cifrová P. García-González J. Kotannal Baby I. Mouillé G. Gineau E. (2018). *Arabidopsis thaliana* plants lacking the ARP2/3 complex show defects in cell wall assembly and auxin distribution. Ann. Bot. 122, 777–789. 10.1093/aob/mcx178 29293873 PMC6215044

[B83] Sambade A. Findlay K. Schäffner A. R. Lloyd C. W. Buschmann H. (2014). Actin-dependent and -independent functions of cortical microtubules in the differentiation of arabidopsis leaf trichomes. Plant Cell 26, 1629–1644. 10.1105/tpc.113.118273 24714762 PMC4036576

[B84] Sampathkumar A. Gutierrez R. McFarlane H. E. Bringmann M. Lindeboom J. Emons A. M. (2013). Patterning and lifetime of plasma membrane-localized cellulose synthase is dependent on actin organization in Arabidopsis interphase cells. Plant Physiol. 162, 675–688. 10.1104/pp.113.215277 23606596 PMC3668062

[B85] Sampathkumar A. Krupinski P. Wightman R. Milani P. Berquand A. Boudaoud A. (2014). Subcellular and supracellular mechanical stress prescribes cytoskeleton behavior in Arabidopsis cotyledon pavement cells. eLife 3, e01967. 10.7554/eLife.01967 24740969 PMC3985187

[B86] Sapala A. Runions J. Routier-Kierzkowska A. L. Das Gupta M. Hong L. Hofhuis H. (2018). Why plants make puzzle cells, and how their shape emerges. eLife 7, e32794. 10.7554/eLife.32794 29482719 PMC5841943

[B87] Savaldi-Goldstein S. Peto C. Chory J. (2007). The epidermis both drives and restricts plant shoot growth. Nature 446, 199–202. 10.1038/nature05618 17344852

[B88] Schindelin J. Rueden C. T. Hiner M. C. Eliceiri K. W. (2015). The ImageJ ecosystem: An open platform for biomedical image analysis. Mol. Reprod. Dev. 82, 518–529. 10.1002/mrd.22489 26153368 PMC5428984

[B89] Schwab B. Mathur J. Saedler R. Schwarz H. Frey B. Scheidegger C. (2003). Regulation of cell expansion by the DISTORTED genes in *Arabidopsis thaliana*: actin controls the spatial organization of microtubules. Mol. Genet. Genomics 269, 350–360. 10.1007/s00438-003-0843-1 12690443

[B90] Smith L. G. Oppenheimer D. G. (2005). Spatial control of cell expansion by the plant cytoskeleton. Annu. Rev. Cell. Dev. Biol. 21, 271–295. 10.1146/annurev.cellbio.21.122303.114901 16212496

[B91] Spitzer M. Wildenhain J. Rappsilber J. Tyers M. (2014). BoxPlotR: a web tool for generation of box plots. Nat. Methods 11, 121–122. 10.1038/nmeth.2811 24481215 PMC3930876

[B92] Staiger C. J. Sheahan M. B. Khurana P. Wang X. McCurdy D. W. Blanchoin L. (2009). Actin filament dynamics are dominated by rapid growth and severing activity in the Arabidopsis cortical array. J. Cell Biol. 184, 269–280. 10.1083/jcb.200806185 19171759 PMC2654301

[B93] Suarez C. Carroll R. T. Burke T. A. Christensen J. R. Bestul A. J. Sees J. A. (2015). Profilin regulates F-actin network homeostasis by favoring formin over Arp2/3 complex. Dev. Cell 32, 43–53. 10.1016/j.devcel.2014.10.027 25543282 PMC4293355

[B94] Szymanski D. B. Cosgrove D. J. (2009). Dynamic coordination of cytoskeletal and cell wall systems during plant cell morphogenesis. Curr. Biol. 19, R800–R811. 10.1016/j.cub.2009.07.056 19906582

[B95] Szymanski D. B. Marks M. D. Wick S. M. (1999). Organized F-actin is essential for normal trichome morphogenesis in Arabidopsis. Plant Cell 11, 2331–2347. 10.1105/tpc.11.12.2331 10590162 PMC144140

[B96] Tian J. Han L. Feng Z. Wang G. Liu W. Ma Y. (2015). Orchestration of microtubules and the actin cytoskeleton in trichome cell shape determination by a plant-unique kinesin. eLife 4, e09351. 10.7554/eLife.09351 PMC4574192 26287478

[B97] Tsukaya H. Tsuge T. Uchimiya H. (1994). The cotyledon: a superior system for studies of leaf development. Planta 195, 309–312. 10.1007/BF00199692

[B98] Ueda K. Matsuyama T. Hashimoto T. (1999). Visualization of microtubules in living cells of transgenic*Arabidopsis thaliana* . Protoplasma 206, 201–206. 10.1007/BF01279267

[B99] Vaškovičová K. Žárský V. Rösel D. Nikolič M. Buccione R. Cvrčková F. (2013). Invasive cells in animals and plants: searching for LECA machineries in later eukaryotic life. Biol. Direct 8, 8. 10.1186/1745-6150-8-8 23557484 PMC3663805

[B100] van der Honing H. S. van Bezouwen L. S. Emons A. M. Ketelaar T. (2011). High expression of Lifeact in *Arabidopsis thaliana* reduces dynamic reorganization of actin filaments but does not affect plant development. Cytoskeleton 68, 578–587. 10.1002/cm.20534 21948789

[B101] van der Kammen R. Song J. Y. de Rink I. Janssen H. Madonna S. Scarponi C. (2017). Knockout of the Arp2/3 complex in epidermis causes a psoriasis-like disease hallmarked by hyperactivation of transcription factor Nrf2. Development 144, 4588–4603. 10.1242/dev.156323 29113991

[B102] van Gisbergen P. A. Li M. Wu S. Z. Bezanilla M. (2012). Class II formin targeting to the cell cortex by binding PI(3,5)P(2) is essential for polarized growth. J. Cell Biol. 198, 235–250. 10.1083/jcb.201112085 22801781 PMC3410418

[B103] Verger S. Chabout S. Gineau E. Mouille G. (2016). Cell adhesion in plants is under the control of putative O-fucosyltransferases. Development 143, 2536–2540. 10.1242/dev.132308 27317803 PMC4958334

[B104] Voigt B. Timmers A. C. Šamaj J. Müller J. Baluška F. Menzel D. (2005). GFP-FABD2 fusion construct allows *in vivo* visualization of the dynamic actin cytoskeleton in all cells of Arabidopsis seedlings. Eur. J. Cell Biol. 84, 95–608. 10.1016/j.ejcb.2004.11.011 16032928

[B105] Wang J. Xue X. Ren H. (2012). New insights into the role of plant formins: regulating the organization of the actin and microtubule cytoskeleton. Protoplasma 249, S101–S107. 10.1007/s00709-011-0368-0 22215231

[B106] Wang J. Zhang Y. Wu J. Meng L. Ren H. (2013). AtFH16, an Arabidopsis type II formin, binds and bundles both microfilaments and microtubules, and preferentially binds to microtubules. J. Int. Plant Biol. 55, 1002–1015. 10.1111/jipb.12089 23802884

[B107] Wang P. Richardson C. Hawes C. Hussey P. J. (2016). Arabidopsis NAP1 regulates the formation of autophagosomes. Curr. Biol. 26, 2060–2069. 10.1016/j.cub.2016.06.008 27451899

[B108] Winter D. C. Choe E. Y. Li R. (1999). Genetic dissection of the budding yeast Arp2/3 complex: a comparison of the *in vivo* and structural roles of individual subunits. Proc. Natl. Acad. Sci. U.S.A. 96, 7288–7293. 10.1073/pnas.96.13.7288 10377407 PMC22078

[B109] Xing H. L. Dong L. Wang Z. P. Zhang H. Y. Han C. Y. Liu B. (2014). A CRISPR/Cas9 toolkit for multiplex genome editing in plants. BMC Plant Biol. 14, 327. 10.1186/s12870-014-0327-y 25432517 PMC4262988

[B110] Yanagisawa M. Zhang C. Szymanski D. B. (2013). ARP2/3-dependent growth in the plant kingdom: SCARs for life. Front. Plant Sci. 4, 166. 10.3389/fpls.2013.00166 23802001 PMC3689024

[B111] Yanagisawa M. Desyatova A. S. Belteton S. A. Mallery E. L. Turner J. A. Szymanski D. B. (2015). Patterning mechanisms of cytoskeletal and cell wall systems during leaf trichome morphogenesis. Nat. Plants 1, 15014. 10.1038/nplants.2015.14 27246881

[B112] Yanagisawa M. Alonso J. M. Szymanski D. B. (2018). Microtubule-dependent confinement of a cell signaling and actin polymerization control module regulates polarized cell growth. Curr. Biol. 28, 2459–2466. 10.1016/j.cub.2018.05.076 30033335

[B113] Yang W. Ren S. Zhang X. Gao M. Ye S. Qi Y. (2011). Bent uppermost internode1 encodes the class II formin FH5 crucial for actin organization and rice development. Plant Cell 23, 661–680. 10.1105/tpc.110.081802 21307285 PMC3077787

[B114] Žárský V. Cvrčková F. Potocký M. Hála M. (2009). Exocytosis and cell polarity in plants - exocyst and recycling domains. New Phytol. 183, 255–272. 10.1111/j.1469-8137.2009.02880.x 19496948

[B115] Zhang X. Dyachok J. Krishnakumar S. Smith L. G. Oppenheimer D. G. (2005). IRREGULAR TRICHOME BRANCH1 in Arabidopsis encodes a plant homolog of the actin-related protein2/3 complex activator Scar/WAVE that regulates actin and microtubule organization. Plant Cell 17, 2314–2326. 10.1105/tpc.104.028670 16006582 PMC1182491

[B116] Zhang C. Mallery E. L. Schlueter J. Huang S. Fan Y. Brankle S. (2008). Arabidopsis SCARs function interchangeably to meet Actin-related protein 2/3 activation thresholds during morphogenesis. Plant Cell 20, 995–1011. 10.1105/tpc.107.055350 18424615 PMC2390748

[B117] Zhang C. Halsey L. E. Szymanski D. B. (2011). The development and geometry of shape change in *Arabidopsis thaliana* cotyledon pavement cells. BMC Plant Biol. 11, 27. 10.1186/1471-2229-11-27 21284861 PMC3042916

[B118] Zhang Z. Zhang Y. Tan H. Wang Y. Li G. Liang W. (2011). Rice morphology determinant encodes the type II formin FH5 and regulates rice morphogenesis. Plant Cell 23, 681–700. 10.1105/tpc.110.081349 21307283 PMC3077795

[B119] Zhang C. Mallery E. Reagan S. Boyko V. P. Kotchoni S. O. Szymanski D. B. (2013a). The endoplasmic reticulum is a reservoir for WAVE/SCAR regulatory complex signaling in the Arabidopsis leaf. Plant Physiol. 162, 689–706. 10.1104/pp.113.217422 23613272 PMC3668063

[B120] Zhang C. Mallery E. L. Szymanski D. B. (2013b). ARP2/3 localization in Arabidopsis leaf pavement cells: a diversity of intracellular pools and cytoskeletal interactions. Front. Plant Sci. 4, 238. 10.3389/fpls.2013.00238 23874346 PMC3709099

