# Supplementary material for: Division of Labor Between Two Actin Nucleators—the Formin FH1 and the ARP2/3 Complex—in Arabidopsis Epidermal Cell Morphogenesis
Source: Front Plant Sci. 2020 Mar 2;11:148. doi: 10.3389/fpls.2020.00148 (PMC7061858; doi:10.3389/fpls.2020.00148)
Supplement: Supplementary file 1 [file DataSheet_1.pdf]

Supplementary Table S1. Primers used in this study (sequences in 5´ to 3´ orientation).

Primer name	Sequence	Purpose
DT1-BsF_FH1g4	ATATATGGTCTCGATTGACACGGCGGCTACTGTCGGGTT	Amplifying gRNA template (with DT2-BsR_FH1g7)
DT1-F0_FH1g4	TGACACGGCGGCTACTGTCGGGTTTTAGAGCTAGAAATAGC	Amplifying gRNA template (with DT2-R0_FH1g7)
DT2-R0_FH1g7	AACACACGACGCAATGCGGAGGCAATCTCTTAGTCGACTCTAC	Amplifying gRNA template (with DT1-F0_FH1g4)
DT2-BsR_FH1g7	ATTATTGGTCTCGAAACACACGACGCAATGCGGAGGCAA	Amplifying gRNA template (with DT1-BsF_FH1g4)
R_FH1_g4g7seq1	AGATAACCGGCGGTTTTGGT	Sequencing *fh1*:CRISPR mutation
