# Supplementary material for: Division of Labor Between Two Actin Nucleators—the Formin FH1 and the ARP2/3 Complex—in Arabidopsis Epidermal Cell Morphogenesis
Source: Front Plant Sci. 2020 Mar 2;11:148. doi: 10.3389/fpls.2020.00148 (PMC7061858; doi:10.3389/fpls.2020.00148)
Supplement: Supplementary file 2 [file DataSheet_2.pdf]

Supplementary Figure S2. Sequence comparison between the 5´end of the *FH1* and *fh1-CRISPR* alleles. Predicted protein sequence is shown in bold, the single base insertion in *fh1-CRISPR* is highlighted in yellow.

```
                 1        10        20        30        40        50
FH1            1 AAAGAAGAAGATAACACAATGCTCTTCTTCTTATTCTTCTTCTACTTACTCTTATCTTCA
fh1:CRISPR     1 ............................................................
FH1            1                   M  L  F  F  L  F  F  F  Y  L  L  L  S  S
fh1:CRISPR     1                   .  .  .  .  .  .  .  .  .  .  .  .  .  .

                61        70        80        90       100       110
FH1           61 TCCTCCGATCTAGTCTTCGCCGACCGTCGTGTACTCCACGAACCATTCTTCCCTATAGAT
fh1:CRISPR    61 ............................................................
FH1           21 S  S  D  L  V  F  A  D  R  R  V  L  H  E  P  F  F  P  I  D
fh1:CRISPR    21 .  .  .  .  .  .  .  .  .  .  .  .  .  .  .  .  .  .  .  .

               121       130       140       150       160       170
FH1          121 TCACCACCACCGTCACCACCATCACCACCACCACTTCCTAAACTACCATTCTCTTCAACC
fh1:CRISPR   121 ............................................................
FH1           41 S  P  P  P  S  P  P  S  P  P  P  L  P  K  L  P  F  S  S  T
fh1:CRISPR    41 .  .  .  .  .  .  .  .  .  .  .  .  .  .  .  .  .  .  .  .

               181       190       200       210       220       230
FH1          181 ACTCCTCCATCTTCATCAGACCCAAATGCTTCTCCTTTCTTCCCTTTATACCCTTCATCT
fh1:CRISPR   181 ............................................................
FH1           61 T  P  P  S  S  S  D  P  N  A  S  P  F  F  P  L  Y  P  S  S
fh1:CRISPR    61 .  .  .  .  .  .  .  .  .  .  .  .  .  .  .  .  .  .  .  .

               241       250       260       270       280       290
FH1          241 CCACCACCACCTTCTCCAGCCTCCTTCGCTTCTTTTCCGGCGAATATCTCATCTCTAATC
fh1:CRISPR   241 ............................................................
FH1           81 P  P  P  P  S  P  A  S  F  A  S  F  P  A  N  I  S  S  L  I
fh1:CRISPR    81 .  .  .  .  .  .  .  .  .  .  .  .  .  .  .  .  .  .  .  .

               301       310       320       330       340       350
FH1          301 GTCCCTCACGCCACTAAATCCCCACCTAACTCCAAAAAACTCCTTATCGTCGCTATCTCC
fh1:CRISPR   301 ............................................................
FH1          101 V  P  H  A  T  K  S  P  P  N  S  K  K  L  L  I  V  A  I  S
fh1:CRISPR   101 .  .  .  .  .  .  .  .  .  .  .  .  .  .  .  .  .  .  .  .

               361       370       380       390       400       410
FH1          361 GCCGTTTCCTCCGCTGCTTTAGTCGCTCTACTTATCGCTTTACTCTATTGGCGAAGAAGC
fh1:CRISPR   361 ............................................................
FH1          121 A  V  S  S  A  A  L  V  A  L  L  I  A  L  L  Y  W  R  R  S
fh1:CRISPR   121 .  .  .  .  .  .  .  .  .  .  .  .  .  .  .  .  .  .  .  .

               421       430       440       450       460       470
FH1          421 AAACGTAACCAAGATCTTAACTTCTCCGATGATAGCAAAACATACACCACCG-ACAGTAG
fh1:CRISPR   421 ....................................................T.......
FH1          141 K  R  N  Q  D  L  N  F  S  D  D  S  K  T  Y  T  T  D   S  S
fh1:CRISPR   141 .  .  .  .  .  .  .  .  .  .  .  .  .  .  .  .  .  V  Q  *

               481       490       500       510
FH1          480 CCGCCGTGTCTACCCTCCTCCTCCGGCAACGGCGCC
fh1:CRISPR   481 ....................................   Signal peptide
FH1          161  R  R  V  Y  P  P  P  P  A  T  A ...   Extracytoplasmic
fh1:CRISPR   161                                        Transmembrane
```
