# Supplementary material for: Division of Labor Between Two Actin Nucleators—the Formin FH1 and the ARP2/3 Complex—in Arabidopsis Epidermal Cell Morphogenesis
Source: Front Plant Sci. 2020 Mar 2;11:148. doi: 10.3389/fpls.2020.00148 (PMC7061858; doi:10.3389/fpls.2020.00148)

Supplementary Figure S3. Mutual calibration of the two methods of measuring cell circularity, size and solidity employed in this study. Each data point was obtained by determining an average value of the indicated parameter from the same set of photos from a single biological replicate of a single genotype. Values determined by the semi-manual ImageJ based approach are plotted on the X axis, those determined by PaCeQuant on the Y axis. The dotted line indicates the expected linear relationship between results of both approaches.

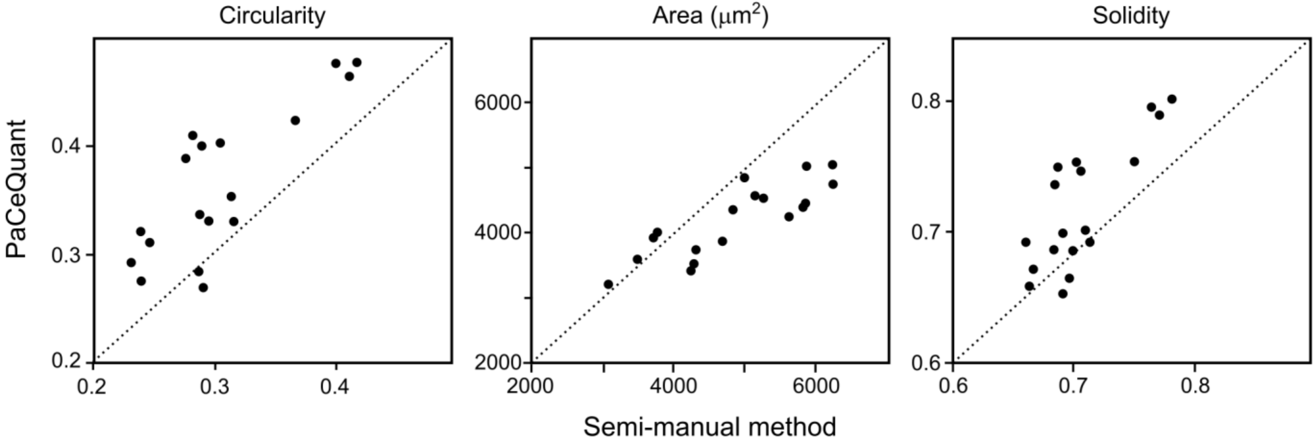

Supplement: Supplementary file 3 [file DataSheet_3.pdf]
