# Supplementary material for: Division of Labor Between Two Actin Nucleators—the Formin FH1 and the ARP2/3 Complex—in Arabidopsis Epidermal Cell Morphogenesis
Source: Front Plant Sci. 2020 Mar 2;11:148. doi: 10.3389/fpls.2020.00148 (PMC7061858; doi:10.3389/fpls.2020.00148)

Supplementary Figure S4. Pavement cell shape in the adaxial epidermis of 14 DAG cotyledons of actin nucleator mutants and wt plants. (A) Representative images of wt, *fh1-1, arpc5* and *fh1-1 arpc5* cotyledon epidermis. (B) Morphometric parameters of cotyledon pavement cells, determined using the semi-manual method (circularity and solidity as measures of cell shape complexity, cell area., aspect ratio). Values labelled by the same letters do not differ significantly ($p < 0.05$).

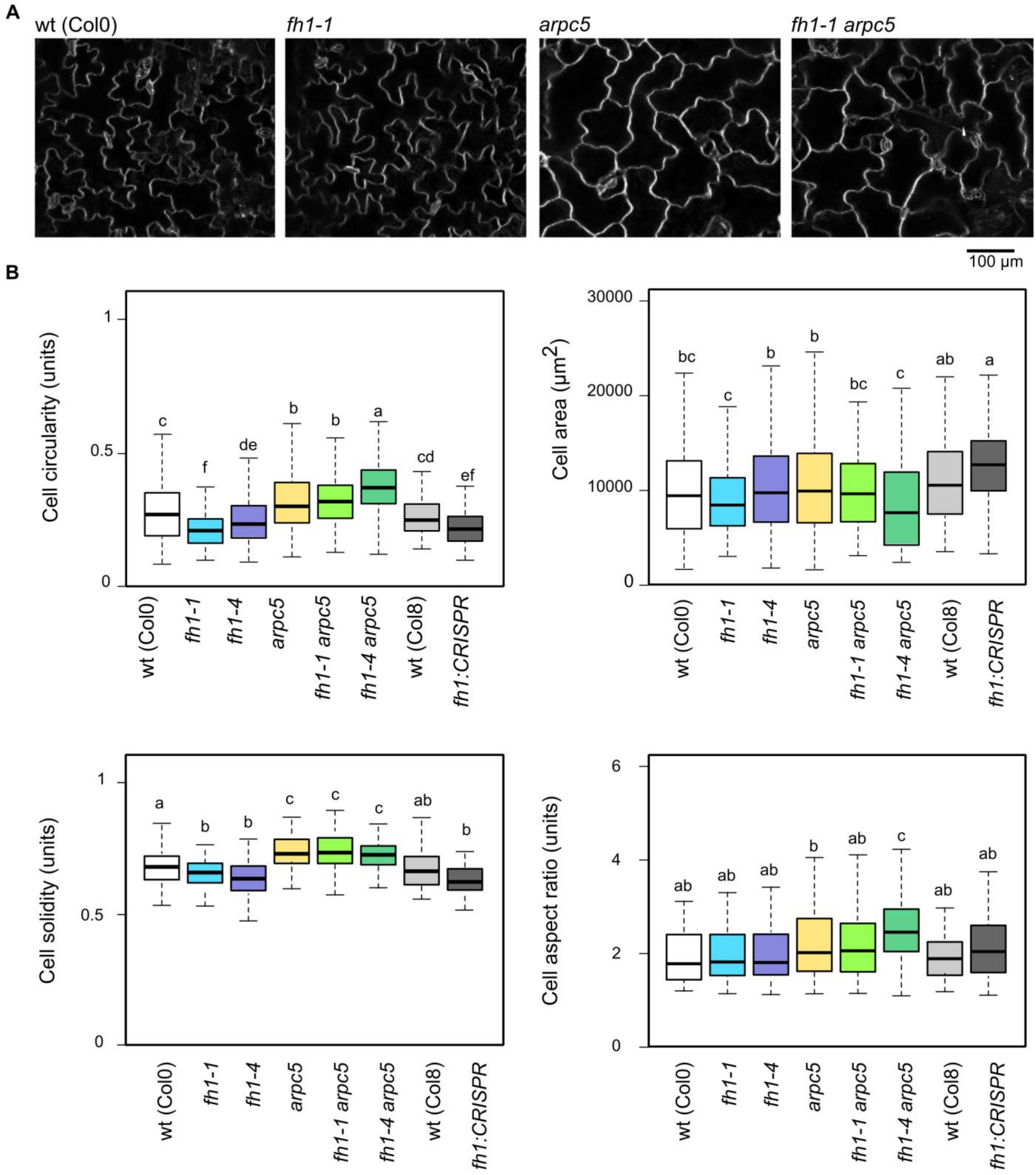

Supplement: Supplementary file 4 [file DataSheet_4.pdf]
