# Supplementary material for: Division of Labor Between Two Actin Nucleators—the Formin FH1 and the ARP2/3 Complex—in Arabidopsis Epidermal Cell Morphogenesis
Source: Front Plant Sci. 2020 Mar 2;11:148. doi: 10.3389/fpls.2020.00148 (PMC7061858; doi:10.3389/fpls.2020.00148)

Supplementary Figure S6. Microtubule organization and dynamics in the adaxial cotyledon epidermis of 5 DAG wt, *arpc5* or *arp2* seedling expressing GFP-TUA6. Quantitative estimates of microtubule bundling (measured as skewness of fluorescence intensity distribution among labelled structures), microtubule network density (measured as pixel occupancy of skeletonized meshwork), microtubule or bundle lifetime (represented by maximum values observed over 120 sec in structures crossing a 2 µm transect) and microtubule or bundle lateral mobility (represented by maximum trajectories observed over 120 sec in structures crossing a 2 µm transect) are shown. Statistical significance of differences is denoted by asterisks (*for $p < 0.05$, **for $p < 0.01$).

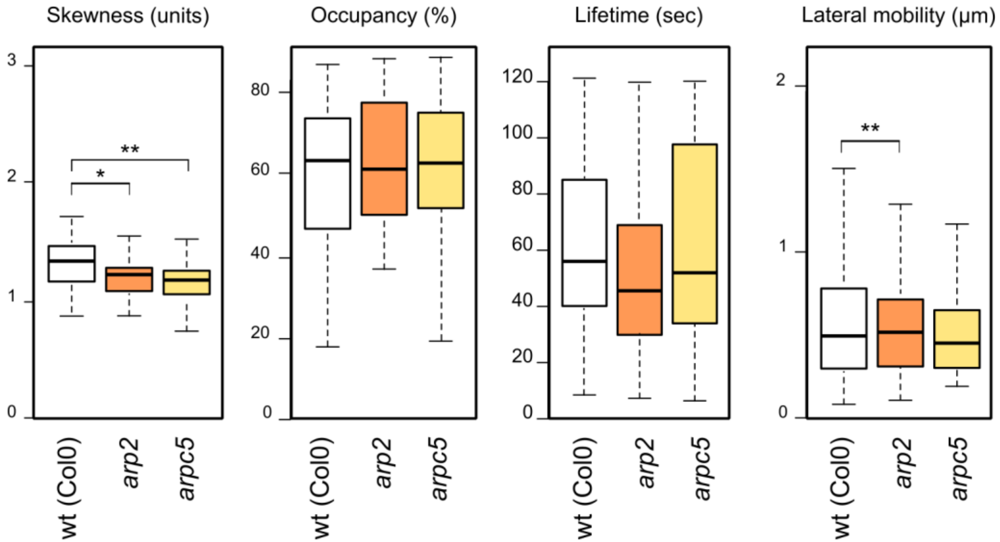

Supplement: Supplementary file 6 [file DataSheet_6.pdf]
