# Supplementary material for: Division of Labor Between Two Actin Nucleators—the Formin FH1 and the ARP2/3 Complex—in Arabidopsis Epidermal Cell Morphogenesis
Source: Front Plant Sci. 2020 Mar 2;11:148. doi: 10.3389/fpls.2020.00148 (PMC7061858; doi:10.3389/fpls.2020.00148)

Supplementary Figure S7. Typical organization of the actin cytoskeleton in developing trichomes on first true leaves of wt plants and actin nucleator mutants expressing Lifeact-GFP. Z-stacks of spinning disc confocal microscopy images are shown.

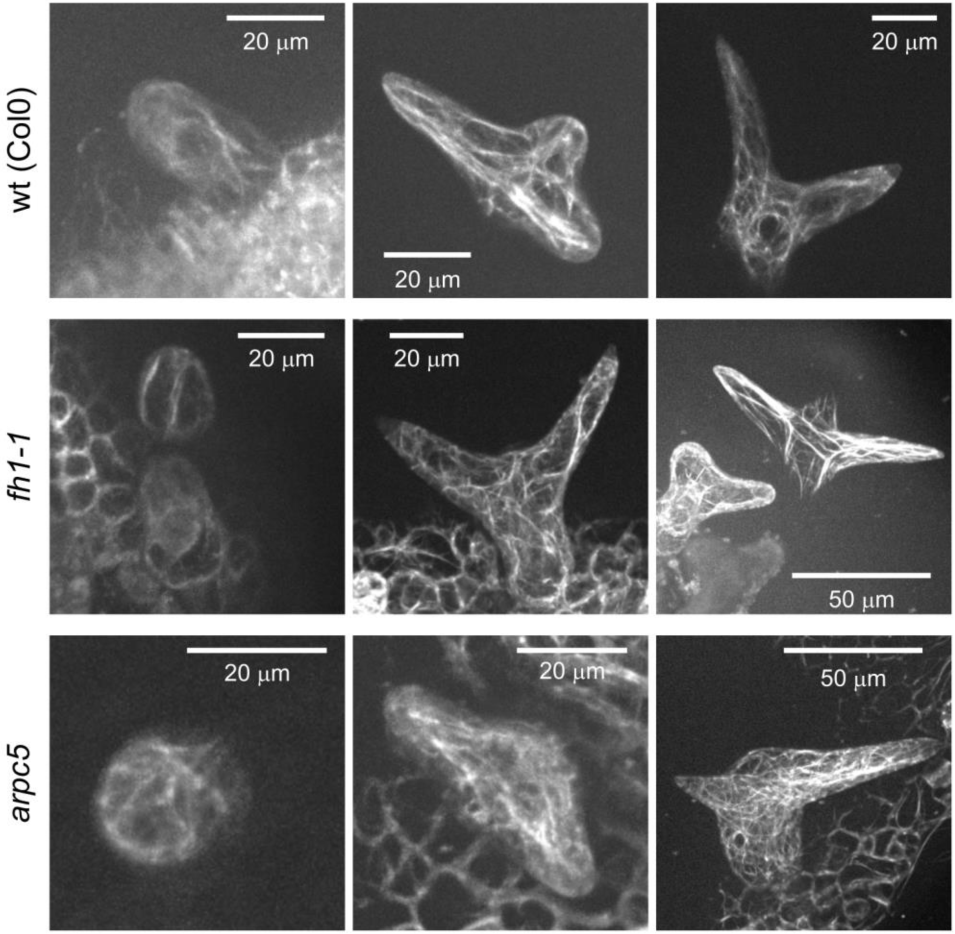

Supplement: Supplementary file 7 [file DataSheet_7.pdf]
