# Supplementary material for: Division of Labor Between Two Actin Nucleators—the Formin FH1 and the ARP2/3 Complex—in Arabidopsis Epidermal Cell Morphogenesis
Source: Front Plant Sci. 2020 Mar 2;11:148. doi: 10.3389/fpls.2020.00148 (PMC7061858; doi:10.3389/fpls.2020.00148)
Supplement: Supplementary file 8 [file DataSheet_8.pdf]

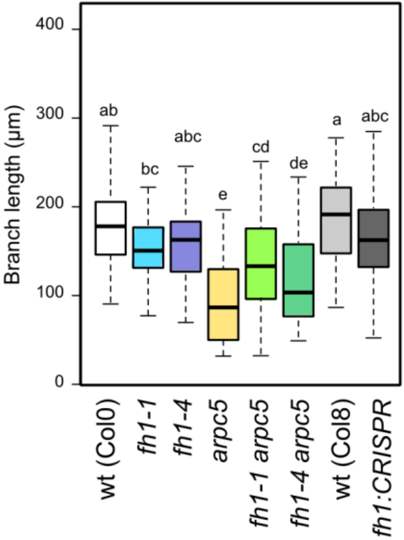

Supplementary Figure S8. Length of terminal branches in 4-branched trichomes of wt plants and actin nucleator mutants. Values labelled by the same letters do not differ significantly ($p < 0.05$).
